# Optimal Design of a Multipole-Electromagnet Robotic Platform for Ophthalmic Surgery

**DOI:** 10.3390/mi14010091

**Published:** 2022-12-29

**Authors:** Ruipeng Chen, David Folio, Antoine Ferreira

**Affiliations:** INSA Centre Val de Loire, 88 Boulevard Lahitolle, 18000 Bourges, France

**Keywords:** electromagnetic actuation system, microrobotic surgery platform, ophthalmic surgery, optimization of magnetic systems, microrobots

## Abstract

The aim of this study was to design a multipole-electromagnet robotic platform named OctoRob. This platform provides a minimally invasive means for targeted therapeutic interventions in specific intraocular areas. OctoRob is capable of generating both appropriate magnetic fields and gradients. The main scientific objectives were: (i) To propose an optimal reconfigurable arrangement of electromagnets suitable for ophthalmic interventions. (ii) To model, design and implement a one-degree-of-freedom robotic arm connected with an electromagnet in order to optimize the generation of magnetic fields and gradients. (iii) To evaluate the magnetic performances of the OctoRob platform, including different tilted angles. The results show that OctoRob platform has great potential to be applied for ophthalmic surgery.

## 1. Introduction

Biomedical magnetic microrobots provide a promising alternative approach for many clinical procedures [1]. To this end, a reliable and effective electromagnetic actuation (EMA) setup should be designed with respect to the medical applications objectives. In previous researches [2,3], the magnetic source generation, the minimum number of electromagnets for an EMA system and the optimization methodology for the configuration of electromagnets have been fully studied for different common applications. This previous work showed that the EMA optimal design is strongly related to the considered application. In particular, most EMA arrangements, such as OctoMag-like and MiniMag-like [4], exhibit different magnetic performances for different coil positions and orientations [2]. However, with a fixed configuration of their coils, OctoMag or MiniMag cannot be easily adapted to different application contexts. Therefore, here, we focus on the optimization and implementation of an EMA platform using the methodology proposed in [3] with the initial motivation of applying it to ophthalmic minimally invasive surgery (MIS) using medical magnetic microrobots that have to achieve various tasks. Through the theoretical analysis of system design, a robotic microrobotic EMA platform named OctoRob with a re-configurable setting was developed.

**Figure 1:**
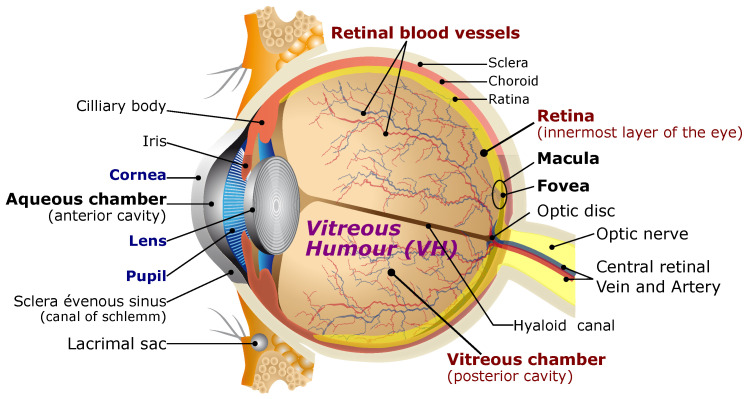
Schematic representation of the human eye.

**Figure 2:**
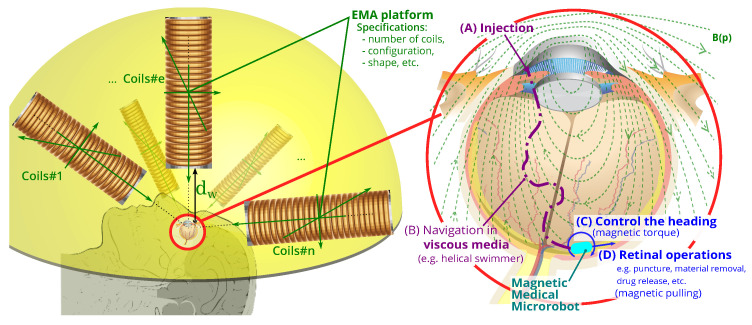
Illustration of the ophthalmic microrobotic MIS system: (**left**) the EMA platform that should respect the geometry of the human head and be arranged in the yellow hemisphere; (**right**) the ophthalmic microrobotic MIS procedure where biomedical mirorobot is injected by a surgeon and controlled by the EMA setup; the generated magnetic field and gradient induce the magnetic torque and pulling force in the medical microrobot for the different steps of the operations.

Many retinal procedures were limited by human performance and perception [5,6,7]. The manipulation of retinal membranes is very delicate, and safe interaction requires forces at best in the order of what the surgeon can receive [5]. The use of microrobots will mitigate traction on the retina, potentially circumventing the necessity to remove some vitreous humor (i.e., perform a vitrectomy) from the eye [4,8,9,10,11]. Figure 1 illustrates the classical anatomy of the human eye. It can navigate in the eye cavity and then perform ophthalmic MIS tasks (material removal, drug delivery, punctures, etc.), and thus has the great potential to revolutionize eye therapy and improve the patient care and recovery. Specifically, a biomedical microrobot can be injected into the retina through a small incision and controlled by manipulating the applied magnetic fields and gradients induced from the developed OctoRob platform, as depicted in Figure 2. OctoRob should be designed accordingly to manipulate efficiently the untethered microrobot with full dexterity. In particular, the biomedical microrobot would have to navigate in the mostly gel-like medium of the vitreous humor (VH). Under such physiological condition, the helical microswimmer is known to be the most efficient propulsion mechanism and thus needs a proper magnetic field [12,13,14,15,16,17]. Then, magnetic force would be required to perform the therapeutic tasks (puncture, peeling, etc.) and thus needs a proper magnetic gradient field. Therefore, generating the appropriate magnetic fields and gradient distribution from the considered OctoRob setup is an important issue that must be investigated with regard to this specific biomedical application.

This paper is devoted to the study of the modeling, design and optimization of an appropriate magnetic robotic platform to provide more effective magnetic fields and gradients on microrobots for performing the given medical tasks—here, an ophthalmic MIS intervention. To that end, the paper is organized as follows. Ophthalmology and ophthalmic surgery challenges and opportunities are first discussed, and the requirements of the considered application are thereby specified. The overall concept of the designed EMA system is represented according to the given specifications stated in [3,18]. A set of electromagnetic coils is firstly investigated, including its magnetic core and its optimal dimensions. Then, the designed coil is evaluated and implemented. Furthermore, a suitable design of the robotic arm is applied. The kinematic mechanism is then analyzed. The robotic arm is integrated in order to optimize the performance of dexterity of magnetic fields and gradients, as introduced in [2,3]. Finally, we describe the building of the designed prototype from the ophthalmic application specifications. The magnetic capability of OctoRob was evaluated to characterize its capability of inducing either good magnetic fields or good gradients.

## 2. Ophthalmic Surgery Specifications

Ophthalmology is the branch of medicine that is concerned with the eye and its diseases. There exist a number of textbooks with in-depth analyses of the eye, such as the work by Snell and Lemp [19]. Similarly, there are numerous conditions of the eye that all require their specific forms of treatments. In particular, the discipline of vitreoretinal surgery includes basically all operations related to the vitreous and to the delicate retina (see also Figure 1), whose health is essential for good vision. Such ophthalmic procedures have great potential to be performed, involving less invasiveness through medical intraocular magnetic microrobots. Retinal surgery requires extremely precise movements and small tool–tissue interaction forces. Otherwise, there is a potential risk of permanent damage (i.e., permanent vision loss) through even small surgical error. For instance, one particularly difficult procedure is the retinal vein cannulation to alleviate its occlusion. Such retinal occlusion is one of the most common causes of vision loss in patients. Its prevalence is 1.6% for adults aged 49 years or older [20]. Various treatment methods for this pathology have been proposed. For instance, intravitreal thrombolysis (thrombolysis, also called fibrinolytic therapy, is the breakdown of blood clots formed in blood vessels) with tissue plasminogen activator injection is the most promising treatment [21]. To do so, thrombolytic enzymes (i.e., clot busting) have to be injected into the tiny occluded vein (diameter of about 100 μm).

Another challenging procedure is the peeling of epiretinal membranes (also called macular pucker). This disease of the eye makes a change in the VH, leading to distortions in the vision, and its contraction can lead to severe vision impairment. The formation of such scar tissue on the retina can have a variety of reasons—age, trauma, idiopathic causes, etc. In order to remove such a membrane, the surgeon can peel it off with micro-forceps. All these retinal procedures involve accurate positioning and force sensing that are at or beyond the sensation and control ability of most surgeons [5,6,7]. For instance, Gupta et al. [5] determined that only approximately 20% of contact events between the surgical tool and the retinal tissue during retinal microsurgery can be felt by the practitioner. This implies that most ophthalmic procedures are likely performed without haptic feedback and the surgeon relies mainly on visual tissue interaction. This lack of haptic feedback could adversely affect surgical outcome, as previous studies have shown that using only visual feedback increases the duration of manual manipulation tasks and reduces precision. Roughly 75% of all forces measured during retinal microsurgery were found to be less than 7.5 mN [5]. Although the forces in the study of Jagtap and Riviere [6] were somewhat higher, there is still substantial evidence that the forces involved during retinal surgery are at or beyond the limits of human perception.

In addition to its complexity, conventional vitreoretinal surgery is an invasive process. During the intervention, different tools are placed in the eye, such as an irrigation line to maintain constant pressure, a light source to improve the visibility and the surgical instrument required to perform the desired task [22]. Furthermore, most procedures that are performed on the retina require a preliminary vitrectomy (i.e., removing some VH) to be able to target the actual disease site. Nevertheless, a vitrectomy is an arduous procedure that requires extreme care. Moreover, the performance is hampered because it is difficult to determine the VH that needs to be removed. Additionally, there is a high risk of complications after the procedure.

Due to the difficulty of accessing to the delicate retina during the MIS procedures, vitreoretinal surgery could be rendered less invasive and safer through the use of intraocular magnetic microrobots. In comparison to larger tools, the small size of such therapeutic agents will mitigate traction on the retina, providing the potential to completely circumvent the necessity to perform a vitrectomy to access the retina. With the goal of enabling less invasiveness and safer retinal operations, and providing an increased level of dexterity desired by clinicians, the designed EMA platform is considered for the magnetic manipulation of a fully untethered and dexterous microrobotic device inside the volume of a human eye.

### 2.1. Ophthalmic Microrobotic MIS

Even the most skilled surgeons have involuntary physiological tremors, which causes most ophthalmic surgeries to be at the peak of human capabilities [5,6,7]. To improve the surgeon’s performance, various robotic technologies have been involved [4,8,9,10,11]. Although most of these current robotic solutions improve the quality of ophthalmic surgery, the invasiveness of the procedure is not fully reduced. The use of untethered magnetic microrobot is a promising alternative to enhance the overall ophthalmic MIS operations.

There is a wide variety of solutions and techniques to magnetically actuate a medical microrobot. The choice of the proper magnetic microrobotic system should be defined with respect to the biomedical application, here for an ophthalmic MIS procedure. The envisioned microrobotic MIS system for eye intervention is shown in Figure 2. First, a magnetic microrobot is injected by the operator through a tiny incision to potentially circumvent the necessity of performing a vitrectomy. The biomedical microrobot consists of magnetic material and can be controlled through the applied magnetic field. Next, the magnetic microrobot is actuated to navigate in the VH that fills the space between the lens and the retina (see also Figure 1). Its workspace is basically filled by a transparent gel-like steady medium with a non-Newtonian rheological property [23,24,25]. In detail, the VH is composed of about ~99% water, ~0.9% salts and ~0.1% of a network of collagen, fibrils and hyaluronanic acid, which all form a scaffold [25]. Collagen is the main structural protein in the extracellular matrix in the various connective tissues in the body. Hyaluronic acid (HA) is an anionic glycosaminoglycan (an animo sugar) that is a major component of synovial tissues and fluid. The collagen concentration in VH is around $40\;\mu g/{cm}^{-3}$ to $120\;\mu g/{cm}^{-3}$, and collagen type II is the most abundant type in the eye. This presence of collagen leads to a gelatinous consistency, and thus the VH has a viscosity 2–4 times greater than that of water ($\rho_{\mathrm{water}}=1\mathrm{mPa}s$) To perform the specified medical tasks, the microrobot must be capable of moving efficiently and reliably in the 3D workspace of the eyeball. Typically, a microrobot is manipulated in fluids in the low-Reynolds-number regime (e.g., $R_{e}\ll 1$), where viscous drag significantly dominates over inertia [26]. In such physiological conditions, helical microswimming is known to be one of the most efficient propulsion mechanisms [12,13,14,15,16,17].

The microrobot is positioned and oriented on the targeted site and then performs the specified ophthalmic tasks, such as targeted therapy and/or material removal (peeling, puncturing, drug delivery, etc.). For instance, to perform a lamina puncture for the treatment of retinal vein occlusion, the biomedical microrobot must navigate to the desired location and be rotated to a given heading to successfully puncture the considered tissue. To realize targeted drug delivery, the microrobot must also navigate to a specific region to control the drug release kinetics and modulate the concentration at the therapeutic window. To do so, the overall motion of the magnetic microrobot in the 3D workspace of the eyeball is simply summarized as its three translations and two rotations—that is, its five degrees of freedom (DOF). The motion of microrobot can be controlled by either the applied magnetic field or its gradient. Basically, the magnetic field produces a magnetic torque $t_{m}$ in order to rotate the microrobot or perform a corkscrew swim or a drilling operation. The magnetic gradient is commonly used to generate a pulling force $f_{m}$ to propel the microrobot or to perform a robotic task (puncture, peeling, etc.).

Finally, as shown in Figure 2, the external EMA platform should be conveniently placed in front of the head at a suitable distance with respect to the eye. The control of the magnetic actuation should precisely and reliably meet the requirements of the ophthalmic MIS procedure. The next section sets out the specifications for the design of such an EMA platform for ophthalmic MIS operation.

### 2.2. Intraocular MIS Operations Requirements

**Figure 3:**
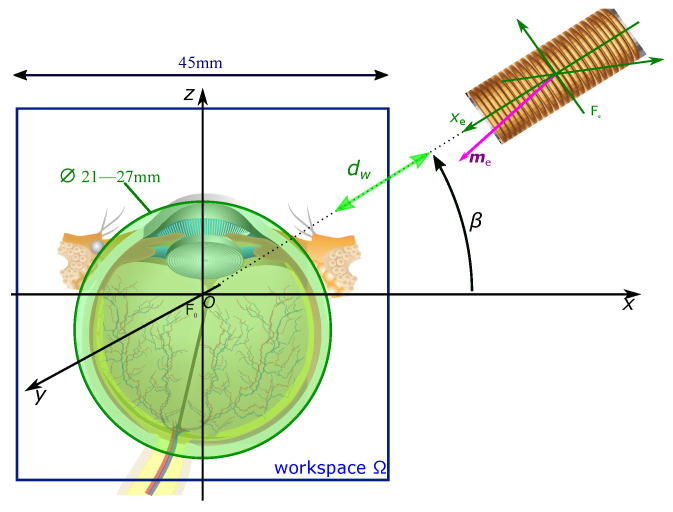
Representation of the workspace with respect to the eye geometry.

First, the geometric constraints of the eyeball and the head should be considered. As reported by Bekerman et al. [27], the size of a human adult eye is about 22 mm to 27 mm; there is not a significant difference among genders or age groups. Hence, the diameter of the considered workspace should be at least 27 mm. However, some extra spaces should be reserved for other eye tissues and the movement of eyeballs. Similarly, the workspace Ω should be defined with respect to the working distance $d_{w}$ between the workspace center *O* and the electromagnet, as reported in Figure 3. From the analysis carried in [2], $d_{w}$ should be sufficiently small to enable strong magnetic field in Ω. Based on these results and requirements, the workspace is defined as a cube of volume of Ω =45 mm×45 mm×45 mm with a working distance $d_{w}$ =65 mm.

In the considered intraocular application, the microrobot be used in the VH to perform the biomedical tasks. Basically, the VH is a complex transparent biofluid that exhibits non-Newtonian rheological properties. Commonly, two different phases are distinguished in the VH: (i) a liquid phase near the center of the eyeball, and (ii) a gelatinous phase near the edges due to the presence of the network of collagen fibrils and hyaluronic acid [23,24,25]. Specifically, Bonfiglio et al. [23,24] report that the kinematic viscosity can be considered to be from $5\times 10^{-6}\;m^{2}\;s^{-1}$ to $8\times 10^{-4}\;m^{2}\;s^{-1}$. The density is in the range of $1.0053\;g\;m^{-3}$ to $1.0089\;g\;m^{-3}$. The VH’s liquid phase has a surface tension of $47.8\;mN\;m^{-1}$, so it behaves like a typical viscoelastic gel, having an elastic region and a delayed elastic region [25]. Therefore, the physiological properties of the medium change significantly, and the EMA platform should adapt the magnetic field and gradient distribution accordingly.

As reported by Amblard et al. [28], a force between 0.1 pN tando 1 pN is enough for moving a micro-object through a moderately dense actin filament network. If the microrobot is made of SPIO material with a magnetization of 50 emu/g and an equivalent diameter of about L ~ 2 μm, a magnetic field magnitude of about ∥B∥=200 mT at least and a magnetic gradient strength of 0.1 mT/m are required. Let us recall that the necessary field intensity decreases or increases following a cubic power of the characteristic length ($L^{3}$) of the magnetic material of the microrobot. Additionally, for some operations, such as human-retinal-vessel puncture, stronger magnetic forces are required. For instance, Dogangil et al. [29] reported some experimental results on the required magnetic forces for puncture tasks with an upper bound of around 10 mN. Their results are in agreement with medical data given by Gupta et al. [5], where it is reported that most puncture forces during vitreoretinal surgery should be below 12.5 mN. Obviously, smaller and sharper microneedles will reduce the required puncture force [30]. Therefore, for a microrobot of a few L ~ 100 mm, the required magnetic field strength could be assumed to be at least about ∥B∥=15 mT, and the magnetic gradient strength at least in the order of 100 mT/m. To summarize, the required amount of magnetic fields and gradients may vary according to the tasks that have to be achieved by the medical microrobot, such as navigating in different VH phases and performing the specified ophthalmic interventions (peeling, puncturing, etc.).

Given these biomedical specifications, the designed EMA platform must provide both the magnetic fields and the gradients necessary to produce efficient magnetic torque $t_{m}$ and force $f_{m}$. The magnetic field distribution is affected by many parameters, including the number of electromagnets or their arrangement, and also by their shape and material properties. Secondly, to allow a reliable and efficient ophthalmic MIS intervention, the characteristic of these fields must be able to adapt to the task in progress. Thereby, the reconfigurable EMA setup fulfilling the application objectives will be designed in detail in the following section using the methodology described in [3].

## 3. OctoRob Platform Design

The effective design of the EMA setup must best meet the requirements for the considered application objectives set out in the previous section. Since the main components of the system are the electromagnetic coils, it is necessary to determine their number, arrangement, material properties, geometry and size.

From the previous investigation [2,31], at least either n=8 stationary electromagnets or n=5 mobile electromagnets sources are required to perform 3D magnetic manipulation in the eyeball. As mentioned in [3], with more coils, the EMA setup will be able to provide stronger, more flexible and less-singular magnetic control. Therefore, we chose to use n=8 electromagnets to design the OctoRob platform.

Next, with the suitable electromagnets set, the first issue is to choose the configuration arrangement of the electromagnets around the human head. Indeed, the chosen configuration must yield the human head geometry while allowing the eyeball as the workspace (see Figure 2 and Figure 3). Commonly, the minimum size of the workspace is restricted by the dimensions of the coils, which are also related to the shortest distance $d_{w}$. Obviously, a larger electromagnetic coil leads to a more powerful magnetic source, but it will make the coils more crowded around the workspace. Furthermore, the larger coils generally require longer distance $d_{w}$. Thus, the maximum diameters of electromagnets can be obtained through their most compact geometric arrangements considering a working distance with a value of $d_{w}$ =65 mm.

From the previous studies, either the OctoMag-like or the MiniMag-like configuration seems to be a proper arrangement solution to fulfill the OctoRob design specifications. In addition, the realized OctoRob system should be adaptable and adjustable in real-time with respect to the microrobotic task. The distance ($d_{w}$) and orientation (β) of each electromagnet are the most important parameters to be adjusted [3]. Specifically, the adaptation of the moving angle β of a mobile coil set makes it possible to favor either the magnetic field or its gradient. That is why a kinematic mechanism should be devised in order to adapt in real-time the orientation of each movable electromagnet.

The reconfigurable electromagnet system is realized by the use of robotic arms. Since the mobile angle β at the end-effector of the robotic arm is the sole parameter, only the kinematic rotation is utilized to achieve dynamic analysis. Thereby, the simulations of the realized robotic EMA system will be further investigated for the estimation of its performance. These different aspects are presented in the following sections.

### 3.1. Modeling of Multipole Electromagnetic Coils System

**Figure 4:**
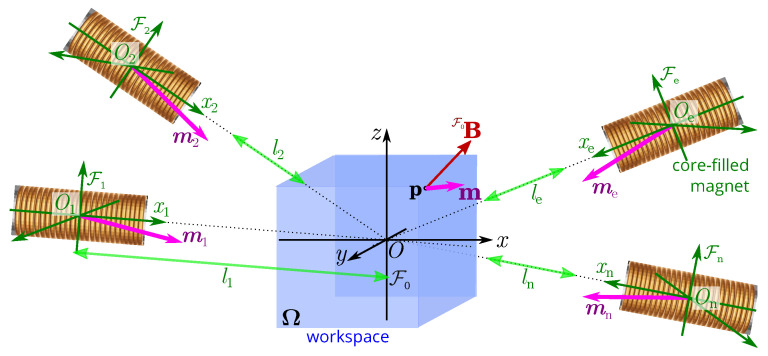
Representation of the multipole-electromagnet system to induce a magnetic field B(p) in the workspace Ω (blue square box). The length $l_{e}$ denotes the distance between the coil center $O_{e}$ and the workspace center *O*.

Three magnetic fields and five magnetic gradient inputs are required to achieve up to 5-DOF control of a microrobot without a singularity [2,32]. Let us consider an overall magnetic field ${}^{F_{0}}B\left(p\right)$ generated by a set of *n* electromagnets at any point p within the workspace Ω, as presented in Figure 4. This global magnetic field is basically the sum of the contributions of all individual electromagnets *e*.

To evaluate the magnetic field $B_{e}\left(p\right)$ induced by the electromagnets *e*, models based either on numerical or analytical approaches are commonly considered. Numerical methods are generally based on the interpolation of the magnetic field obtained either by the finite element method (FEM) or through an experimental measurement of the field $B_{e}$ [4]. Such numerical methods provide high-precision magnetic field calculations, but they are very time-consuming. To speed up the computations, analytical methods commonly based on dipole approximation or even on elliptical integrals are used [4,33,34,35]. Hybrid approaches using a map of the magnetic field obtained from FEM and fitting of an analytical model can be also considered [4]. The choice of method usually results in the best balance between speed and accuracy. Here, we assume that the magnetic field $B_{e}\left(p\right)$ of each point p in Ω is approximated by the magnetic point-dipole model. In the workspace, as shown in Figure 4, the point-dipole model indicating the magnetic field ${}^{F_{e}}B_{e}$ of the coils *e* w.r.t. its own frame $F_{e}\left(O_{e}:x_{e},y_{e},z_{e}\right)$ is expressed as:

(1) $${}^{F_{e}}B_{e}\left(p\right)=\frac{\mu_{0}}{4\pi {\left|p\right|}^{3}}\left(\frac{3\left(m_{e}\;\cdot \;p\right)}{{\left|p\right|}^{2}}-m_{e}\right)$$

with $m_{e}$ being the equivalent magnetic dipole moment of the electromagnetic coil *e* for a unit current input ($i_{e}=1$).

The magnetic field ${}^{F_{e}}B_{e}\left(p\right)$ should be expressed in the reference frame $F_{0}\left(O:x,y,z\right)$ by the homogeneous transformation:

(2) $${}^{F_{0}}B_{e}\left(p\right)={}^{F_{0}}T_{F_{e}}\times {}^{F_{e}}B_{e}\left(p\right)$$

where the homogeneous transformation matrix is basically defined as:

$${}^{F_{0}}T_{F_{e}}=\left(\begin{array}{cc} {}^{F_{0}}R_{F_{e}} & {}^{F_{0}}t_{F_{e}}\\ 0 & 1 \end{array}\right)$$

where ${}^{F_{0}}R_{F_{e}}$ is the rotation matrix and ${}^{F_{0}}t_{F_{e}}$ represents the translation matrix regarding the reference frame $F_{0}$. Therefore, the global magnetic field distribution in the workspace generated by a set of *n* electromagnetic coils can be superimposed as:

(3) $${}^{F_{0}}B\left(p\right)=\sum_{e=1}^{n}{}^{F_{0}}B_{e}\left(p\right)=\left(\begin{array}{ccc} {}^{F_{0}}{\tilde{B}}_{1}\left(p\right) & \ldots & {}^{F_{0}}{\tilde{B}}_{n}\left(p\right) \end{array}\right)i={}^{F_{0}}B\left(p\right)i$$

with $i={\left(i_{1},i_{2},\ldots ,i_{n}\right)}^{⊤}$ being the applied electrical currents. This relation (3) shows that the induced magnetic field ${}^{F_{0}}B\left(p,i\right)$ and its gradient in the workspace can be adjusted by controlling the flowing currents i. When the electromagnet is dynamically moved, either the magnetic field or the gradient can be favored.

### 3.2. Electromagnetic Coil Design

The core-filled electromagnet has been selected for the OctoRob system. The core-filled electromagnet is able to generate a stronger magnetic field than the air-filled electromagnet, since the magnetic core can concentrate the magnetic field. Basically, the magnetic material, the shape and the geometry of the core are the key design parameters of the electromagnet.

#### 3.2.1. Magnetic Core

**Figure 5:**
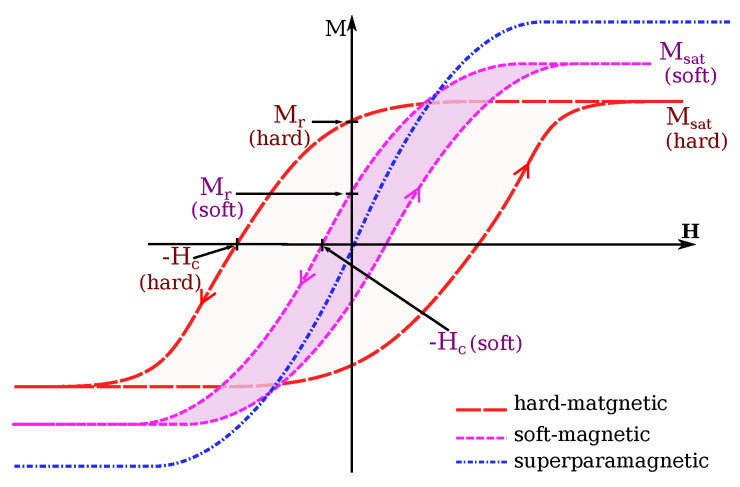
Typical hysteresis curves for ferromagnetic materials. The intercepts $H_{c}$ and $M_{r}$ are the intrinsic coercivity and magnetization remanence.

The presence of a magnetic core involves some non-linearity and coupling between the electromagnets in the magnetic field distribution. Furthermore, different loss effects occur when the flowing current is varying in the coils, such as winding, eddy currents and hysteresis losses. Therefore, the choice of its material is an important issue. Basically, soft-ferromagnetic material should be favored [36]. Indeed, to select an efficient magnetic core material, its permeability, saturation magnetization ($M_{sat}$, A/m) and coercivity ($H_{c}$, A/m) are the main relevant characteristics, as illustrated in Figure 5. The higher permeability and saturation magnetization are preferred for flux confinement and focusing. A low coercivity is important for high-frequency applications and to reduce the core losses. Table 1 reports properties of some common soft-magnetic materials. Moreover, some additional constraints should be also considered in the choice of the core material. For instance, to deal with mechatronic constraints, its weight may come an essential issue. Obviously, the cost of the material is also a significant factor in the final choice.

The shape of its tip should then be determined. Commonly, flat-faced, rounded and sharp-faced magnetic cores have been investigated by researchers [39,40]. In particular, if the tip of the magnetic core is not flat but pointed or round, the magnetic field is nonuniform and severely distorted. In contrast, the use of sharp-faced allows the generation of higher magnetic field and a larger field gradient near the electromagnet. For instance, the effect of core tip geometry on a magnetic-field projection is evaluated and reported by Kummer [39] with the FEM analysis. The analysis indicates that the softer magnetic material is packed up to the very edge of the coil. The more induced magnetic field will be emitted, and electromagnet will generate a stronger magnetic field. For our application objective with a working distance of $d_{w}$ =65 mm, a flat-faced tip (or equivalent cylindrical tip) can make an electromagnet generate the strongest and the most uniform magnetic field in a workspace of Ω =45 mm×45 mm×45 mm. However, the flat-faced tip could cause more crowded space when the n=8 electromagnetic coils are arranged together around the workspace. It is especially clear that the induced magnetic field will be enhanced when magnetic core increases its volume. Thereby, with sufficient space for the electromagnet to move, the core-filled electromagnet can be enlarged to increase the induced magnetic field strength. The electromagnet dimensions will be discussed hereafter.

#### 3.2.2. Optimal Electromagnets Sizing

**Figure 6:**
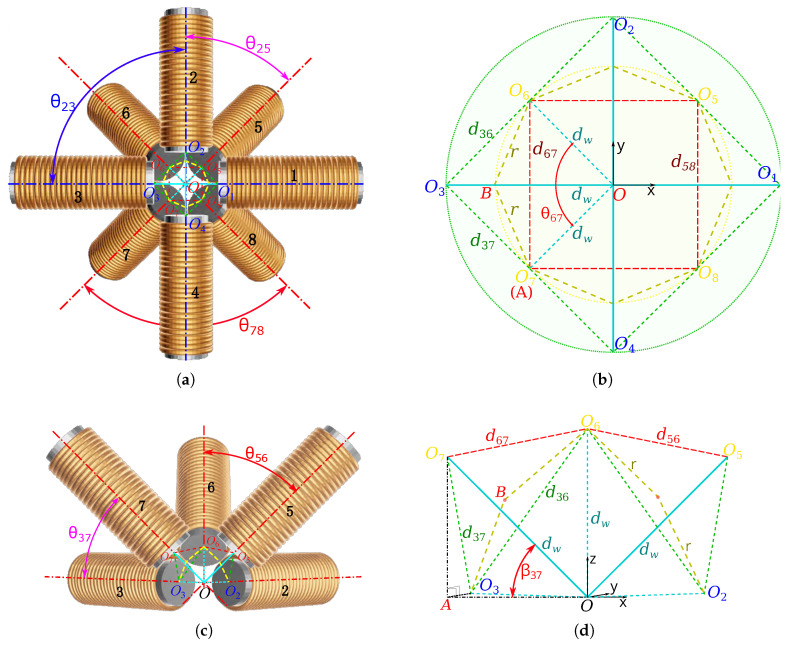
Representation of the geometric arrangement of the 3D n=8 electromagnetic coils: (**a**) top views; (**c**) side views where three electromagnetic coils are removed to show clearly internal space; (**b**,**d**) the geometry between the electromagnets’ center $O_{e}$. The plain lines depict the in-plane lengths, whereas dashed lines represent the out-of-plane ones.

Having suitable electromagnet dimensions is important for generating the necessary magnetic field and gradient in a limited workspace Ω. The requirement for magnetic manipulation in the compatible intraocular procedure leads to a working distance of about $d_{w}$ =65 mm. As mentioned in Figure 6, the different coils of the OctoRob platform are divided in two sets: (i) four stationary electromagnets: e=i=1…4; and (ii) four mobile coils: e=j=5…8. These two sets are arranged around a common *z* axis with an azimuth angle $\alpha_{e}=45{}^{\circ}$ (e=1…8) and point to the common center *O*. We consider that the n=8 electromagnets are identical with a cylindrical geometry and an external radius *r* (the external radius encompass the core, the coil winding and eventually the cooling part). They are initially considered placed to get the maximum value s $r_{\max}$ of their dimensions. Specifically, the largest $r_{\max}$ is obtained when all coils are closely contacted with their neighboring coils. In our case, any mobile coils are in contact with all its adjacent coils (either a mobile or stationary ones). However, the stationary coil set is in contact only with the neighboring mobile coils, but not with the other (stationary) coils. For instance, the stationary coil i=1 touches the $j_{1}=5$ and $j_{2}=8$ ones, whereas mobile coil 5 is in contact with 2, 6, 1 and 8.

**Figure 7:**
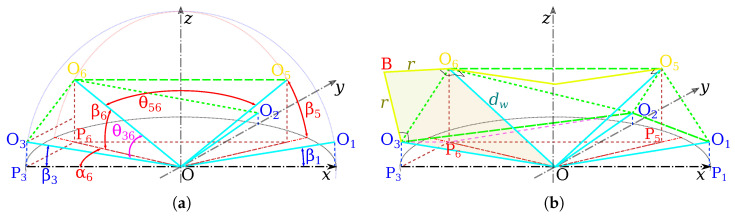
Representations of (**a**) the angles and (**b**) lengths’ definitions.

Figure 6 represents the geometric view of the n=8 electromagnetic coils, where the cyan lines denote the distance $d_{w}$ $=OO_{e}$ from the tip center $O_{e}$ of the electromagnet *e* to the workspace center *O*; the yellow lines represent its external radius *r*; and the green lines indicate the distances between the core center $O_{e}$ and the adjacent contacting electromagnets. First, due to the axisymetric arrangement, the distances $d_{i_{1}i_{2}}=O_{i_{1}}O_{i_{2}}$ between two neighboring stationary coils $i_{1}$ and $i_{2}$ are identical; in the same way, all distances $d_{j_{1}j_{2}}=O_{j_{1}}O_{j_{2}}$ between two adjacent moving coils $j_{1}$ and $j_{2}$ are equal. Next, each electromagnet has a cylindrical face of radius *r*. It can be shown that *r* is tangent to the sphere of radius $d_{w}$, and they are on a same plane for the two contacting coils, and then $OO_{e}=$$d_{w}$ and *r* intersect at right angles (90°), as represented in Figure 6b,c and Figure 7. As all coils share the same radius *r* and working distance $d_{w}$, for stationary coils i=1…4 and their adjacent mobile coils j=5…8, the distances $d_{ij}=O_{i}O_{j}$ all have the same length. ($O_{i}O_{j}$: possible cases are $O_{1}O_{5}$, $O_{2}O_{5}$, $O_{2}O_{6}$, $O_{3}O_{6}$, $O_{3}O_{7}$, $O_{4}O_{7}$, $O_{4}O_{8}$ and $O_{1}O_{8}$). Thus, a mobile coil j=5…8 and its two adjacent stationary coils $i_{1}$ and $i_{2}$ form an isosceles triangle $O_{i_{1}}O_{j}O_{i_{2}}$, and in the same way $O_{j_{1}}O_{i}O_{j_{2}}$ also form an isosceles triangle. Following the same reasoning, it can be seen that all angles $\theta_{j_{1}j_{2}}$ between two adjacent mobile coils $j_{1}$ and $j_{2}$ are identical ($\theta_{j_{1}j_{2}}$: possible cases are $\theta_{56}$, $\theta_{67}$, $\theta_{78}$ and $\theta_{85}$); and similarly, every angle $\theta_{ij}$ between a mobile coil *j* and a neighboring stationary coil *i* has the same value. ($\theta_{ij}$: possible cases are $\theta_{15}$, $\theta_{25}$, $\theta_{26}$, $\theta_{36}$, $\theta_{37}$, $\theta_{47}$, $\theta_{48}$ and $\theta_{18}$).

To determine the maximum value $r_{\max}$ for every angle $\beta_{i}$ and $\beta_{j}$, let us define the projection $P_{e}$ of the center $O_{e}$ of the electromagnet *e* in the xy-plane, as represented in Figure 7a. As one can see, each angle $\theta_{ij}$ is defined as:

$$\theta_{ij}=2\arcsin\left(\frac{d_{ij}}{2d_{w}}\right)$$

with the length $d_{ij}=O_{i}O_{j}=\sqrt{P_{i}P_{j}^{2}+{\left(O_{j}P_{j}-O_{i}P_{i}\right)}^{2}}$, where

(4) $$\begin{array}{cc} P_{i}P_{j} & =\sqrt{OP_{i}^{2}+OP_{j}^{2}-2\left(OP_{i}\right)\left(OP_{j}\right)\cos\alpha_{j}}\;\left(\mathrm{here}\;\alpha_{j}=45{}^{\circ}\right)\\ O_{i}P_{i} & =d_{w}\sin\beta_{i}\\ O_{j}P_{j} & =d_{w}\sin\beta_{j}\\ OP_{i} & =d_{w}\cos\beta_{i}\\ OP_{j} & =d_{w}\cos\beta_{j} \end{array}$$

Similarly, the angle between two adjacent mobile coils $j_{1}$ and $j_{2}$ is derived as:

$$\theta_{j_{1}j_{2}}=2\arcsin\left(\frac{d_{j_{1}j_{2}}}{2d_{w}}\right)$$

where $d_{j_{1}j_{2}}=O_{j_{1}}O_{j_{2}}=\sqrt{OP_{j_{1}}^{2}+OP_{j_{2}}^{2}}$. If all mobile coils *j* have the same moving angle $\beta_{j}$, then we obtain $d_{j_{1}j_{2}}=\sqrt{2}d_{w}\cos\beta_{j}$ from (4). When a stationary coil *i* touches a mobile coil *j*, their radii must be:

$$r_{ij}=\frac{d_{ij}}{2\cos\left(\theta_{ij}/2\right)}=\frac{d_{ij}}{2\sqrt{1-{\left(\frac{d_{ij}}{2d_{w}}\right)}^{2}}}$$

and when a mobile coil $j_{1}$ is in contact with a mobile coil $j_{2}$, their radii are defined as:

(5) $$r_{j_{1}j_{2}}=\frac{d_{j_{1}j_{2}}}{2\cos\left(\theta_{j_{1}j_{2}}/2\right)}=\frac{d_{j_{1}j_{2}}}{2\sqrt{1-{\left(\frac{d_{j_{1}j_{2}}}{2d_{w}}\right)}^{2}}}$$

Since all radii are equal, we get: $r_{max}=r_{ij}=r_{j_{1}j_{2}}$. Hence, it can be shown that the maximal admissible radii are obtained when

$$2-\sqrt{2}\cos\left(\beta_{i}\right)\cos\left(\beta_{j}\right)-2\sin\left(\beta_{i}\right)\sin\left(\beta_{j}\right)=2\cos{\left(\beta_{j}\right)}^{2}$$

**Figure 8:**
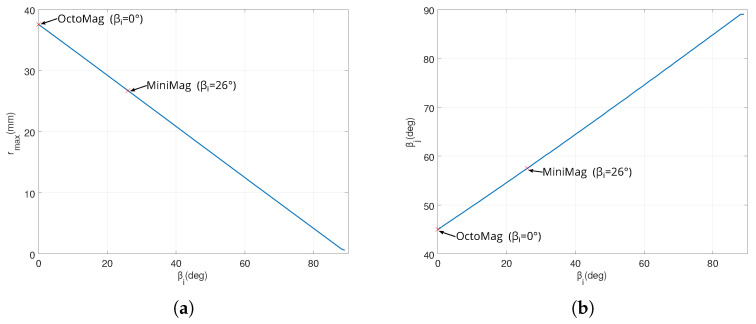
Evolution of (**a**) admissible radius $r_{max}$ and (**b**) the mobile angle $\beta_{j}$ when the contact constraints are satisfied.

Figure 8 shows the evolution of the admissible radius $r_{max}$ and of mobile angle $\beta_{j}$ when the contact constraints are satisfied. For instance, when the OctoMag configuration is considered [4], $\beta_{i}=0$ for the stationary coil set leads to $\beta_{j}=45{}^{\circ}$ for the moving coil set (or the upper set equivalently). Obviously, $d_{ij}\;=\;d_{j_{1}j_{2}}\;=\;d_{w}$ can be computed from the above equations that provides the maximum coil dimensions. Thus, when the $d_{w}$ is set to 65 mm, the radius of $r_{max}=37.5278\;\mathrm{mm}$ can be derived.

#### 3.2.3. Coil Implementation

**Figure 9:**
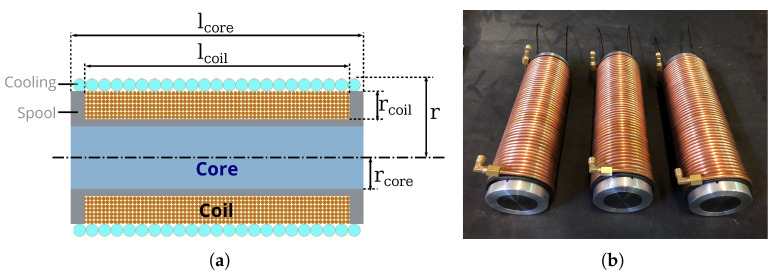
(**a**) The electromagnet’s shape, with its core, coil winding and cooling part. The spool part allows containing the winding and separating it from the core. (**b**) The implemented electromagnet prototype.

As specified, a cylindrical electromagnetic coil is considered for this platform, as depicted in Figure 9. In such case, the magnetic performance can be approximated using a solenoid model. Indeed, the magnetic field strength at the end of an infinite solenoid corresponding to an electromagnet *e* is given by [41] through applying the Ampère’s law:

(6) $$∥B_{e}∥=\mu \frac{i_{e}N}{2l}$$

with $i_{e}$ being the electric current, *l* the length of the solenoid and *N* the number of turns of the coil winding.

To maximize the magnetic field strength, two parts of the electromagnet can be optimized: (i) the core and its permeability performance μ; and (ii) the coil winding. To do so, when the maximum allowable electromagnet size $r_{max}$ with respect to the spatial arrangement is determined, its remaining dimensions could be estimated easily. Kummer [39] has proposed an electromagnet design that takes into consideration further constraints. Specifically, they have shown that the proper length to radius ratio of the core should be at least $l_{core}/r_{core}\geq 8$ to fit properly the above solenoid characteristics. From their results, it appears that a core with a radius of about $r_{core}\approx 20\;\mathrm{mm}$ and a length $l_{core}=10\times r_{core}$ enables reliable magnetic field performance.

Next, the coil thickness should be determined. From the infinite solenoid (6), the best magnetic performance is achieved when the number of turns (*N*) is maximized. However, the winding of coil is limited by the current density that could cause a safety issue. Basically, from transformer design, without cooling, it is shown that the current density should not exceed $J_{max}=3A/{\mathrm{mm}}^{2}$. Nevertheless, to enable a strong magnetic field, the current $i_{e}$ must be high enough. Therefore, the parameter of $J_{max}=3A/{\mathrm{mm}}^{2}$ normally requires the wire to be made of a larg cross-section of conductor, which will limit the number of turns *N*. In order to increase $i_{e}$ together with a great *N* in a limited room, a cooling system will be mandatory. Different strategies can be envisioned for the cooling part, such as using a fan, a radiator vent or a water/coolant system. Classically, using a circulating coolant is the most effective technology for cooling a system.

From these considerations, we chose the following design parameters to implement each electromagnet. The core was composed of low-carbon steel (C35, ThyssenKrupp AG, France) with $r_{core}=21\;\mathrm{mm}$ and $l_{core}=240\;\mathrm{mm}$. Aluminum (a paramagnetic material that is essentially unaffected by the magnetic fields; Aluminum EN AW-2017a-en 573-3, ThyssenKrupp AG, France) spool is added to separate the core and the coil, and to keep the winding in the given space. This spool has an inner length of $l_{coil}=210\;\mathrm{mm}$ and a thickness of 1 mm. The coil is composed of winding of six layers of copper wire (copper is a diamagnetic material, and thus is repelled by the magnetic field; Enameled copper wire, cl 200 degrees, grade 2. APX, France) of diameter of 1.6 mm, leading to a thickness of 9.6 mm and a number of turns N=787. Finally, a cooling unit is placed around the coil winding. The cooling system consists of a circulating coolant liquid flowing through a copper tube with a diameter of 4 mm. All these elements lead to an electromagnet with an overall size of r=35.6 mm, and an electromagnet prototype has been realized and is shown in Figure 9b.

#### 3.2.4. Coil Performance Evaluation

The methodology used in the simulation is based on the assumption that the point-dipole model (1) properly approximates the magnetic field distribution. The assumption is validated in this section.

**Figure 10:**
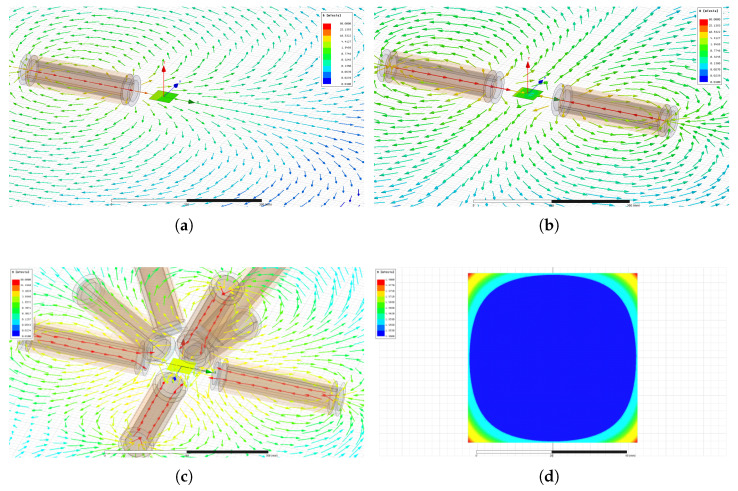
Magnetic field distribution using FEM: (**a**) a single- and (**b**) a dual-electromagnet system; and (**c**,**d**) the eight-electromagnet arrangement with $\beta_{i}=0{}^{\circ}$ and $\beta_{j}=45{}^{\circ}$. (**d**) The magnetic field magnitude within the workspace.

**Figure 11:**
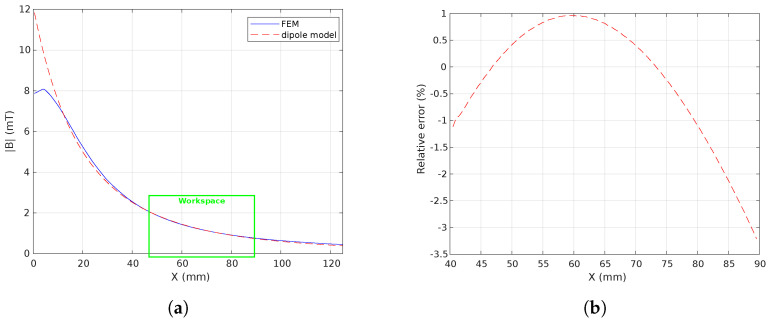
Comparison of simulation results for the FEM and point-dipole model along the *x* axis: (**a**) the magnetic field magnitude where the green box delimits the range of workspace, and (**b**) the relative error within the workspace.

FEM modeling using ANSYS^®^ Maxwell (https://www.ansys.com/products/electronics/ansys-maxwell accessed on 25 November 2022) software has been performed. Hence, the specified electromagnetic coils were modeled and simulated through finite element analysis (FEA). Figure 10 shows some simulation results where a current of 1A/turn is flowing in each electromagnet. Figure 10a,b illustrate the magnetic field B where the workspace is surrounded by a single and a pair of electromagnet(s), respectively. Likewise, Figure 10c,d present the magnetic field distribution of the retained n=8 electromagnets’ configuration where the stationary coil set has an angle $\beta_{i}=0$ and the mobile coil set has an angle $\beta_{j}=45{}^{\circ}$. Figure 11 shows the comparison between the FEM results and the point-dipole results along the *x* axis. Figure 11b reports the relative error on the magnetic field magnitude within the workspace. It appears that the point-dipole model is a convenient method to approximate the magnetic field distribution with a relative error less than |3.5|% in the OctoRob workspace.

### 3.3. Design of the Robotic Arm

The previous motivations and application specifications led to making the OctoRob platform reconfigurable. This makes it possible to sometimes favor the magnetic field and the torque, and sometimes the magnetic field gradient and the traction/thrust force. To achieve this, we propose the design of a robotic arm mechanism for controlling electromagnet orientation in the following.

#### 3.3.1. Robotic Arm Mechanism Description

**Figure 12:**
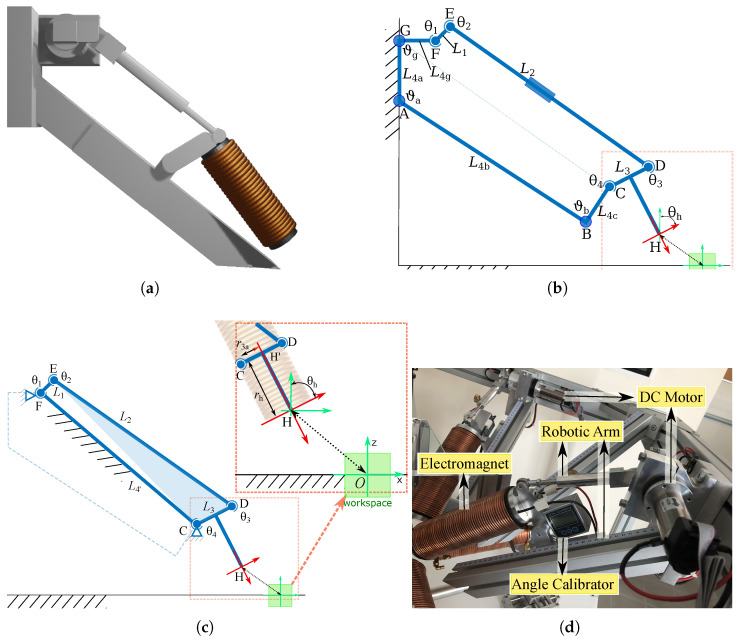
Representation of the robotic arm: (**a**) CAD illustration of the concept, (**b**,**c**) the kinematics chain with the four links $L_{1}$–$L_{4}$ and joints $\theta_{1}$–$\theta_{4}$ and (**d**) the realized 1-DOF robotic arm.

The OctoRob platform must be able to move the mobile coils independently; i.e., their polar angles $\beta_{j}$ should be changed dynamically. A basic solution for actuating the robotic arm is to use simple DC motors. However, its placement must be studied to limit magnetic field interference and prevent the electromagnetic compatibility (EMC) issue. To overcome this problem, the moving coils are placed at the end-effector of a robotic arm, as illustrated in Figure 12. Based on these considerations, and as only one DOF is required, we have chosen to use a well known four-bar linkage mechanism. The four-bar linkage architecture allows reliable 1 DOF motion feature, higher energy efficiency, good rigidity, less link inertia and compact drive systems.

Figure 12b depicts the kinematic chain of the designed four-bar linkage. Specifically, $L_{1}$ is the crank, $L_{2}$ is the coupler and $L_{3}$ is the rocker. In addition, the frame $L_{4}$ is decomposed in four parts: $L_{4a}$, $L_{4b}$, $L_{4c}$ and $L_{4g}$. Each link or part $L_{i}$ has a length $r_{i}$. In particular, the lengths $DE=r_{2}$ and $CH^{\prime }=r_{3a}$ can be easily adjusted manually to modify the kinematics of the electromagnet. The lengths $GA=r_{4a}$ and $AB=r_{4b}$ could be also modified to handle smaller or bigger electromagnetic coils if it is necessary. Once fixed and calibrated, the links’ geometry of the OctoRob platform cannot vary and would lead to the simplified kinematics chain shown in Figure 12c. Table 2 presents some calibrated parameters of the robotic arm. With the proposed specifications, the robotic arms are realized and installed with electromagnets, DC motors and angle calibrators, as represented in Figure 12d.

Next, a motor actuates the joint $\theta_{1}$, which rotates the following links $L_{2}$–$L_{3}$ and the other joints $\theta_{2}$–$\theta_{4}$. Then, the electromagnet fixed to the link $L_{3}$ is able to rotate with $\theta_{h}$.

#### 3.3.2. Kinematic Analysis

As mentioned in Section 3.1, the magnetic field ${}^{F_{0}}B\left(p\right)$ can be expressed in the reference frame $F_{0}$ linked to the workspace center by using the homogeneous transformation, as in Equation (2). Similarly, the input angle $\theta_{1}$ is transferred to the output angle $\theta_{h}$ through applying transformation matrices. For more details of analysis of its definition of transformation, please see Appendix A.

The position of the point *G* is fixed, and we assume that its location is known with respect to the reference frame $F_{0}$ as ${}^{F_{0}}G={\left(x_{g},y_{g},z_{g},1\right)}^{⊤}$ in homogeneous coordinates. Next, when $L_{4a}-L_{4c}$ are set to a given value, the position $C={\left(x_{c},y_{c},z_{c},1\right)}^{⊤}$ of the joint $\theta_{4}$ is fixed and can be determined in $F_{0}$ as:

$${}^{F_{0}}C={\mathrm{Rot}}_{y}\left(\vartheta_{b}\right){\mathrm{Trans}}_{z}\left(r_{4c}\right){\mathrm{Trans}}_{x}\left(r_{4b}\right){\mathrm{Trans}}_{z}\left(-r_{4a}\right)\;{}^{F_{0}}G\;$$

where ${\mathrm{Rot}}_{y}\left(\vartheta_{b}\right)$ denotes the pure rotation operation along the *y* axis with angle $\vartheta_{b}$, and ${\mathrm{Trans}}_{z}\left(r_{4c}\right)$ indicates a pure translation along the *z* axis with the displacement $r_{4c}$.

Similarly, the location of $E={\left(x_{e},y_{e},z_{e},1\right)}^{⊤}$ of the joint $\theta_{2}$ can be expressed in $F_{0}$ as follows:

$${}^{F_{0}}E={\mathrm{Rot}}_{y}\left(\theta_{1}\right){\mathrm{Trans}}_{z}\left(r_{1}\right){\mathrm{Trans}}_{x}\left(r_{4d}\right)\;{}^{F_{0}}G$$

Through the transformation operations, the position of the end-effector ${}^{F_{0}}H$, together with the point ${}^{F_{0}}H^{\prime }$, can be determined by:

$$\begin{array}{cc} {}^{F_{0}}H^{\prime } & ={\mathrm{Rot}}_{y}\left(\theta_{3}\right){\mathrm{Rot}}_{y}\left(\vartheta_{b}\right){\mathrm{Trans}}_{z}\left(r_{3}\right){\mathrm{Trans}}_{z}\left(r_{4c}\right){\mathrm{Trans}}_{x}\left(r_{4b}\right){\mathrm{Trans}}_{z}\left(-r_{4a}\right)\;{}^{F_{0}}G\;\\ {}^{F_{0}}H & ={\mathrm{Rot}}_{y}\left(\pi /2\right){\mathrm{Rot}}_{y}\left(\theta_{3}\right){\mathrm{Rot}}_{y}\left(\vartheta_{b}\right){\mathrm{Trans}}_{z}\left(r_{h}\right){\mathrm{Trans}}_{z}\left(r_{3}\right){\mathrm{Trans}}_{z}\left(r_{4c}\right){\mathrm{Trans}}_{x}\left(r_{4b}\right)\\ \; & \times {\mathrm{Trans}}_{z}\left(-r_{4a}\right)\;{}^{F_{0}}G \end{array}$$

The orientation $\theta_{h}$ of the end-effector can be then determined by the obtained $\theta_{3}$, since the surface of electromagnet is parallel to $L_{3}$; that is: $\theta_{h}=\theta_{3}$. Therefore, both position and orientation parameters of the end-effector can be computed.

## 4. Implementation of the OctoRob Prototype

**Figure 13:**
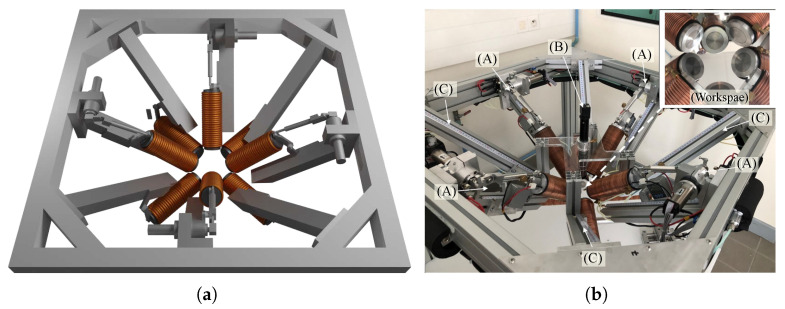
OctoRob design. (**a**) CAD representation of the EMA platform and (**b**) experimental prototype: (A) 1-DOD mobile robotic arms; (B) optical microscope with a CCD camera; (C) stationary robotic arms.

From the design specifications, we fabricated the OctoRob platform illustrated in Figure 13. It is composed of four mobile robotic arms to control the mobile electromagnets’ orientation and four stationary electromagnets.

**Figure 14:**
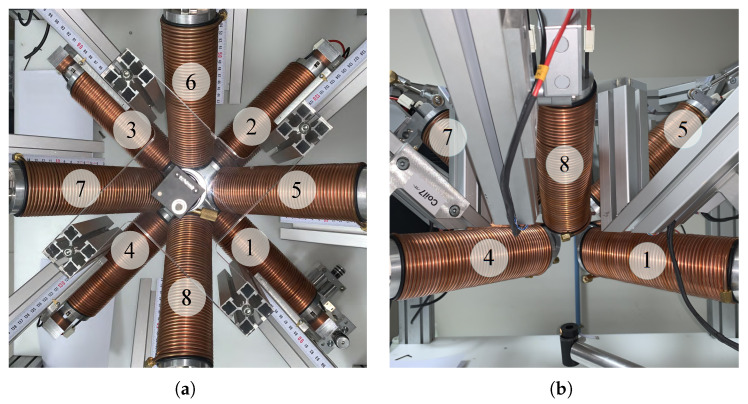
Photograph of the (**a**) top-view and (**b**) sideview of OctoRob prototype.

During the design process, we paid particular attention to the rigidity of the setup due to the long fixed and moving parts, which have direct consequences on micromanipulation accuracy of the microrobotic tool. To deal with these constraints, the robotic arms are rigidly fixed to the frame reference structure to avoid mechanical vibrations to be transmitted to the end-effectors. Furthermore, mechanical deformations of robotic arms linked to external forces have been mechanically reinforced. When the robotic platform has been assembled, the robotic arms axes are calibrated in order to ensure that a unique point *O* is settled at the center of the workspace. The calibration process is based on a laser pointing system where eight laser spots point towards the same focusing point *O* with several-micrometer accuracy. The geometrical design rules have been respected during the design stage in order to have a semi-hemispherical workspace of 45 mm × 45 mm × 45 mm. As shown in the inset of Figure 13b, the limits of semi-hemispherical workspace are prone of any collisions: (1) the optical microscope has direct access to the workspace to provide a top-view of the eyeball, (2) the magnetic coils do not contact each other and (3) the orientations of the mobile coils are limited by mechanical stops. Figure 14 shows closed-views of the OctoRob prototype. In the current configuration, the stationary coils have their polar angles fixed to $\beta_{i}=0$ as the OctoMag setup. If necessary, these polar angles can be modified to other configurations, for instance, to $\beta_{i}=26{}^{\circ}$ as the MiniMag configuration.

**Figure 15:**
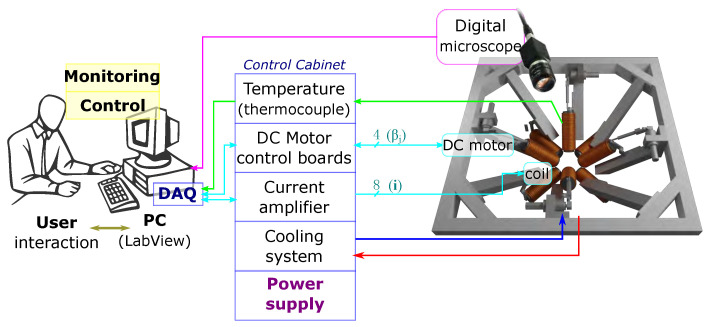
Synoptic of OctoRob platform architecture.

**Figure 16:**
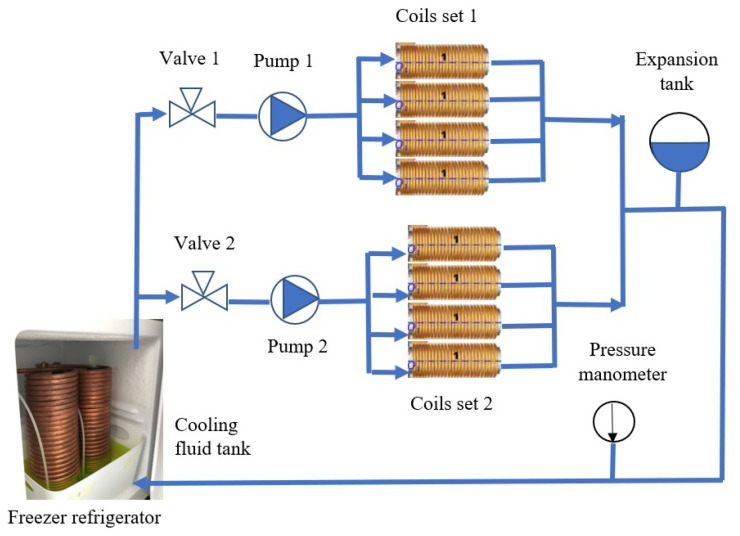
Cooling system for temperature control of electromagnets.

Figure 15 shows the overall system architecture of the OctoRob platform, which is divided into power, driving, cooling and sensing units. The current for the eight electromagnetic coils is sourced through custom-designed switched amplifiers with a maximum current of 10 A per channel and controlled through two data acquisition (DAQ) cards (NI PCI-6229, NI) with 12-bit resolution. The current flowing through each channel is controlled through PID controllers (Epos2 50/2 Maxon) within a range of −10 A to 10 A. However, heat dissipation poses the limit to the maximum achievable magnetic field generated by each electromagnet. We developed a custom cooling system capable of circulating a coolant through the copper tubing wrapped around each coil, as shown in Figure 16. The temperature of coil windings is monitored through temperature thermocouple sensors (−40° up to +100 °C Radial lead, UK) integrated at the center of the coil between the spool and the winding.

The four mobile robotic arms are actuated by DC motors (MDP DCX32L, Beynost, France). A stationary vision system provides visual feedback of the top view of the workspace. The vision system is composed of an optical microscope (M Plan APO SL 100X, Edmund Optics, Barrington, IL, USA) connected to a digital CMOS camera delivering images and real-time video. The working distance of 90 mm with a limited depth-of-field of few tens of mm is used to image microrobots, along with a frame size of 640×480 pixels. Since the interior of the human eye is externally observable by optical microscopy, computer vision algorithms have been developed for intraocular localization using the OpenCV library. The entire system is controlled through a LabView^®^ software (http://www.ni.com/labview/ accessed on 25 November 2022) environment connected to Matlab^®^ (http://fr.mathworks.com accessed on 25 November 2022) by a single computer with an Intel^®^ Core 4 Quad CPU, 3.0 GHz.

## 5. Evaluation of OctoRob

The configuration of the OctoRob system was designed in regard to the considered application—that is, an ophthalmic MIS procedure. To evaluate its performance, further simulations and characterization were carried out.

### 5.1. Magnetic Field and Gradient of the OctoRob Platform

To evaluate the magnetic performance of the OctoRob, we used the average $\langle \varphi \rangle$ and the uniformity γ(φ) metrics defined as follows [2]:

(7) $$\begin{array}{cc} \langle \varphi \rangle & =\frac{1}{N}\sum_{p\in \Omega }^{N}\varphi \left(p\right) \end{array}$$

(8) $$\begin{array}{cc} \gamma (\varphi ) & =1-\frac{1}{2N\langle \varphi \rangle }\sum_{p\in \Omega }^{N}\left|\varphi \left(p\right)-\langle \varphi \rangle \right|\;\;(\mathrm{in}\;\%) \end{array}$$

where *N* is the number of samples and φ is either the magnetic field or its gradient.

As represented in Figure 14, the four stationary electromagnets (e=1,2,3,4) are considered on the common xy plane and behave similarly to Maxwell coils pairs. The left four mobile electromagnets (e=5,6,7,8) are actuated by robotic arms described in Section 3.3. Different cases are investigated hereafter.

**Figure 17:**
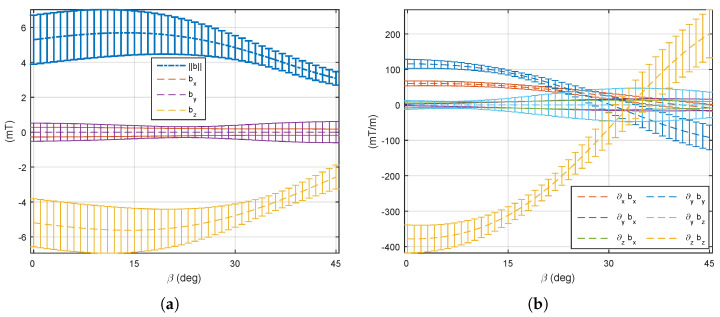
The averages $\langle \varphi \rangle$ of the (**a**) magnetic fields and (**b**) its magnetic gradients with four moving electromagnets rotating with angle β∈[0°,45°]. The error bar shows the standard deviation of the value.

**Figure 18:**
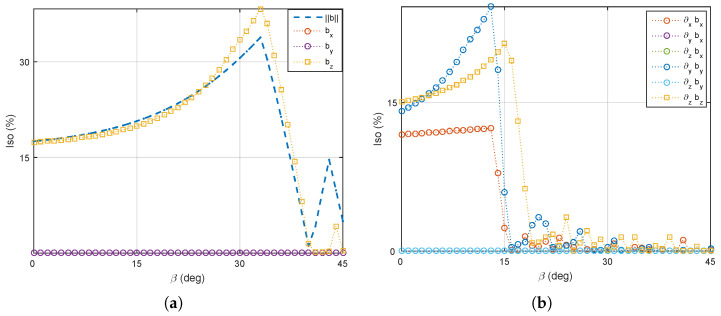
The uniformity γ(φ) of (**a**) magnetic fields and (**b**) its magnetic gradients with four moving electromagnets rotating with angle β∈[0°,45°].

**Figure 19:**
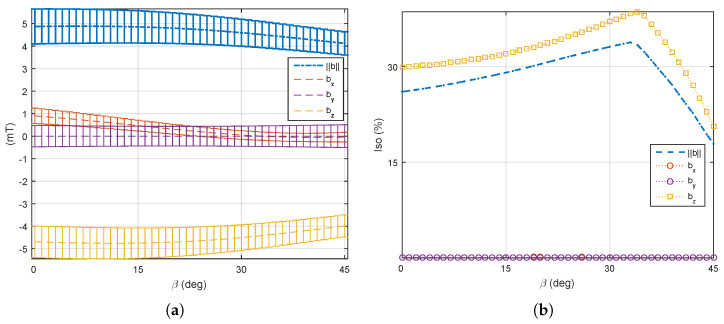
One mobile electromagnet (coil 5) is rotating, and the others are set to β=33°: (**a**) the average and (**b**) the uniformity of the magnetic field.

**Figure 20:**
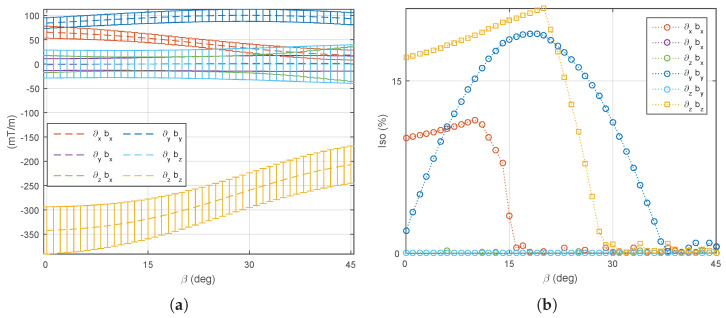
One mobile electromagnet (coil 5) is rotating, and others are fixed to β=14°: (**a**) the average and (**b**) the uniformoty of magnetic gradients.

**Figure 21:**
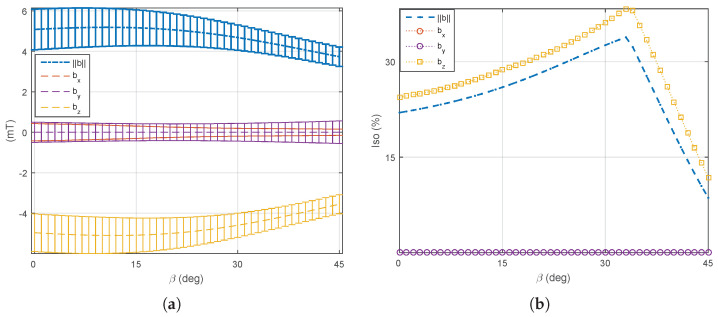
Two mobile electromagnets (dual coils 5 and 7) are rotating and the other coils set to β=33°: (**a**) the average and (**b**) the uniformity of the magnetic field.

**Figure 22:**
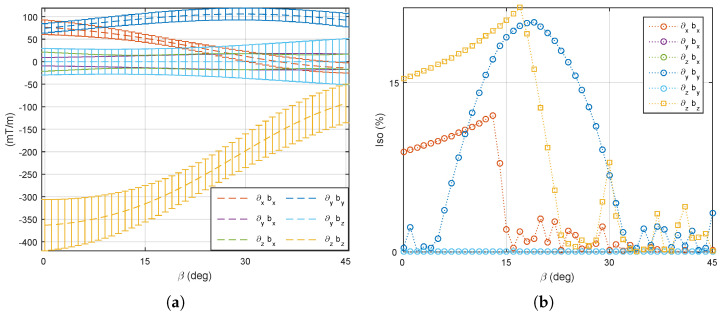
Two mobile electromagnets (dual coils 5 and 7) are rotating and the other coils are set to β=14°: (**a**) the average and (**b**) the uniformity of the magnetic gradient.

(1) The four mobile coils’ rotation: We first consider that the four mobile electromagnets (e=5,6,7,8) are operated in the same way: each mobile coil is rotating together with the same angle β. On this basis, the optimal angle β is investigated to perform different manipulation tasks using either the magnetic field or its gradient. Figure 17 and Figure 18 show the evaluation of the average (7) and uniformity (8) metrics of the magnetic field and its gradient for the rotating angle β∈[0°,45°]. From these results, two distinguishing behaviors appear: (i) the magnetic field is significant enough and more uniform for a mobile angle of around β=33°, whereas (ii) a strong and uniform magnetic gradient appears at β=14°. Therefore, these results demonstrate the need to control either the magnetic field or its gradient by simply adjusting the orientation of certain electromagnets of the OctoRob platform to get a reliable field in the workspace.

(2) A single mobile coil’s rotation: Here we investigate the influence of having a single electromagnet (e=5) allowed to rotate, whereas the other mobile coils (e=6,7,8) are fixed. The four stationary electromagnets (e=1,2,3,4) remain unchanged in the xy plane. As mentioned, for β=33°, the magnetic field is more efficient. Thus, at first the mobile coils are fixed to 33°, and only one coil is rotating. Figure 19 describe the averages strength and the uniformity of the magnetic field in the workspace. Although the magnetic field strength does not change significantly with the rotating angle, a more uniform magnetic field appears at around 35°. Secondly, the magnetic gradient field remains more efficient when β=14°. Figure 20 illustrates the average strength and their uniformity of the magnetic gradients within the workspace. Figure 20a reveals that the magnetic gradient becomes stronger when the mobile angle is below 20°. Moreover, to get more uniform magnetic gradients, it requires rotating the single coil in the range of 5° to 25°. Thus, the mobile angles β∈[5°,20°] can be used for the more effective force control on magnetized microrobots.

(3) Two mobile coils’ rotation: Commonly, electromagnetic coils are used in pairs. Hence, a pair of mobile coils that are opposite each other (see coil 5 and coil 7 in Figure 14) are here allowed to rotate with the same angle β∈[0°,45°], while the other mobile coils remain stationary. In such a case, the performance of a medical EMA system can be adapted by one pair of robotic arms. As previously described, the unchanged mobile coils (e=5,7) are firstly fixed to 33° to favor the magnetic field. Figure 21 reports the performance metrics of the magnetic field, when only a pair of opposite electromagnets is rotating. Next, the fixed mobile coils (e=6,8) are set to 14° to favor the magnetic gradient. Figure 22 illustrates the corresponding performance indicators of the magnetic gradient. As we can see, the influence of the mobile electromagnet pair is quite similar to the result of one single mobile electromagnet.

### 5.2. System Characterization

**Figure 23:**
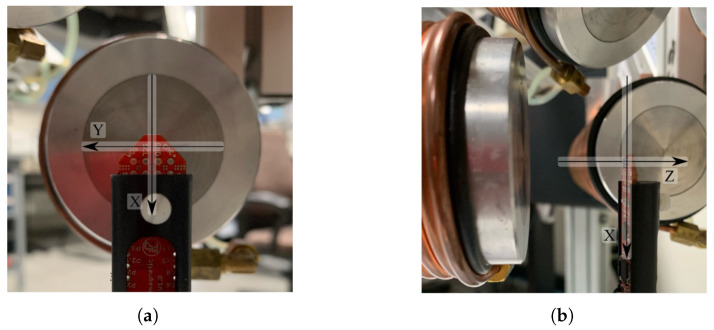
The definitions of the magnetic directions *x*, *y* and *z* of the sensor TLE493D-W2B6. (**a**) The front view of the magnetic sensor; (**b**) the side view of the magnetic sensor.

**Figure 24:**
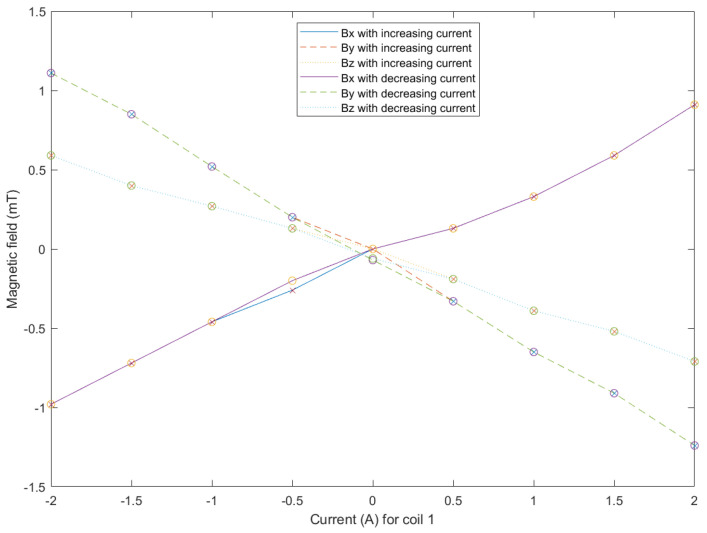
Hysteresis curve for the magnetic field at the center of the workspace as a function of current (−2 A to 2 A) actuating coil 1. Shape x in the curve denotes the data for increasing current, and shape o denotes the data for decreasing current.

The conceptual design of OctoRob is based on the assumption that the use of high-performance soft-magnetic material in the coils cores leads to linear behavior regarding the input currents. Thus, the core of the coil is operated within its linear region (see also Figure 5). To assess this assumption, a magnetic sensor (TLE493D-MS2GO, Infineon) was placed in the center of workspace frame $F_{0}$, as in Figure 23. For the coil e=1, Figure 24 shows the measured magnetic field. These results confirm that the electromagnetic coil works in the linear region. The evaluated linear coefficients are reported in Table 3.

**Figure 25:**
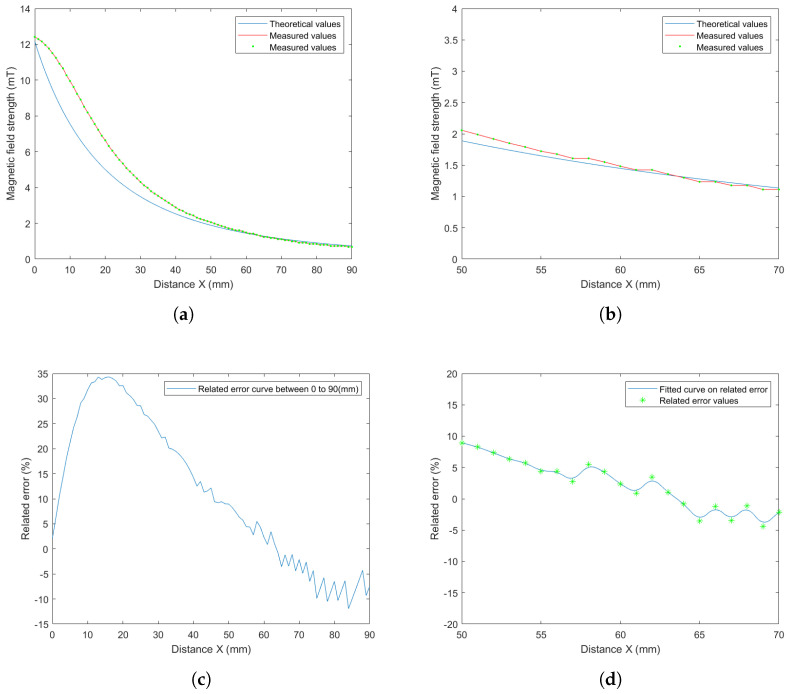
Comparison of theoretical values and measured values of the magnetic field: (**a**) Magnetic field strength along the symmetry axis (*x* axis) of the electromagnet, and comparison of theoretical values and measured values. The detected locations were selected to be from 0 to 90 mm for the measurements and (**b**) the detected locations were selected to be from 50 to 70 mm for the measurements. (**c**,**d**) the relative error within the workspace. The detected locations were selected to be from 0 to 90 mm and from 50 to 70 mm for the measurements. (**a**) Computed values and measured data. (**b**) Computed and measured data in a given small range of distance. (**c**) The relative error within the workspace. (**d**) The relative error within the selected region.

As mentioned above, a unit-current field map must be constructed for each of the electromagnets. An analytical model was chosen to fit the magnetic field data obtained by a finite element method for the unit-current contributions. The point-dipole model, Equation (1), was derived to apply the computation of magnetic fields. Figure 25 shows the magnetic field strength along the *x* axis of the electromagnet *e* computed from Equation (1) and measured with the magnetic sensor. As one can see, the theoretical values obtained using the point-dipole model become close to the measured values for locations between 50 and 70 mm from the coil center, as reported in Figure 25b. When the working distance $d_{w}$ is adjusted to between 50 and 70 mm, the relative error is less than 10%, as presented in Figure 25d This reinforces the choice of the distance $d_{w}$ =65 mm resulting from the specifications indicated in Section 2.2.

**Figure 26:**
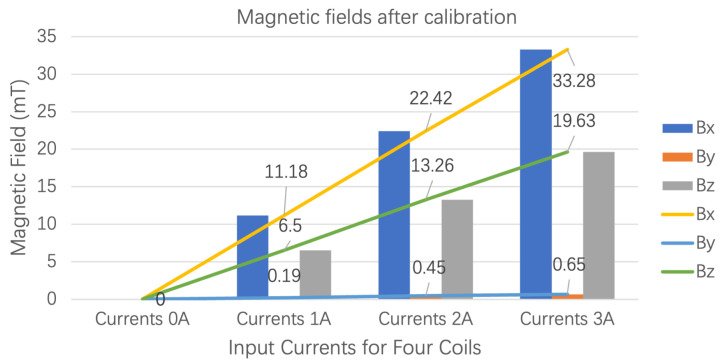
Measured magnetic fields after the calibrations in the workspace center.

After calibration, Figure 26 presents that the magnetic fields generated by the OctoRob platform behave linearly with respect to the input currents flowing through the electromagnets (four coils e=1,2,3,4); currents from 0 to 3 A were applied.

### 5.3. Discussion

The OctoRob platform was designed based on the previous analysis of different EMA arrangements [2]. Globally, it appears that most EMA systems exhibit different magnetic performances for different positions and orientations of their coils. Hence, the built robotized OctoRob system comprises two sets of coils: four fixed electromagnets and four mobile electromagnets. The results indicate that a high moving angle (around β=33°) is most suitable for generating a strong and uniform magnetic field. A most strong and uniform magnetic gradient field can be only achieved at low moving angles, β<20°. Since the magnetic fields induce the torque and the force is commonly generated by magnetic gradients, we demonstrated that the designed OctoRob system provides versatile modes for effective control of biomedical microrobots in different situations. For example, the helical microswimmer could be propelled through the VH using a rotating magnetic field; then, through magnetic gradient manipulation it could perform therapeutic tasks, such as targeted therapy and/or material removal (peeling, puncture, drug delivery, etc.). Therefore, the developed platform would assist surgeons in difficult retinal ophthalmic MIS procedures. Any similar applications where both magnetic fields and gradients are important aspects, and where the hemispherical arrangement is practical, can also be considered. For example, OctoRob can be placed above certain parts of a limb (knee, arm, etc.) or over some organs (liver, kidney, etc.).

Furthermore, the three considered cases, discussed in Section 5.1, provide results that are similar, leading to two interesting aspects. First, we can roughly choose the most simple strategy—for instance, only one electromagnet moving by a robotic arm. However, to achieve more precise and more efficient trajectory tracking or a large magnetic force or torque, more mobile electromagnet should be considered. Secondly, the results also suggest that the moving coils do not necessarily need to be accurately controlled to enable either a strong magnetic field or a gradient capability.

## 6. Conclusions

This paper presents the development of a novel robotized OctoRob system with an initial motivation of improving ophthalmic procedures with untethered biomedical magnetic microrobots. Previous studies have shown that the designed EMA platform must be defined with respect to the considered application. Hence, from the analysis of the specificities of ophthalmic operations, the OctoRob specifications were defined. The proposed EMA system includes four static electromagnets and four robotized electromagnets, allowing ir sometimes to favor the magnetic field and sometimes its gradient. The coil sizing and implementation were performed to provide a balance between the workspace size and the magnetic field performance. Different moving cases of the mobile electromagnets were evaluated. The evaluation of OctoRob’s magnetic capability shows that the mobile electromagnets allow a variety of magnetic field distributions. Specifically, a low angle β leads to stronger and more uniform magnetic gradient distribution, whereas the most uniform magnetic field is produced near a high mobile angle β. Furthermore, the system’s calibration was completed to match the designed model closely. The results of system characterization prove that the implemented platform works in the linear region of the generated electromagnetic fields regarding the input currents. The proposed mathematical model of magnetic field generation was also validated through the calibration process. Therefore, the reconfigurable OctoRob platform allows providing more versatility, maneuverability and flexibility to the magnetic manipulation of biomedical microrobots to help surgeons in difficult ophthalmic surgeries. In the future, model trials regarding ophthalmic applications will be performed with this platform, and the performances of OctoRob will be further investigated and improved.

## Figures and Tables

**Table 1:** Typical properties of some soft-magnetic materials. (Adapted from [37,38,39]).

Material	Saturation (A/m)	Remanence (T)	Coercivity (A/m)	Relative Permeability ($\mu_{r}$)	Density ($\mathrm{kg}/m^{3}$)
Cobalt (Co)	$2.4\times 10^{6}$	0.5	795.7	250	890
Iron (Fe)	$3.1\times 10^{6}$	1.3	79.5	5000	7874
Nickel (Ni)	$5.1\times 10^{5}$	0.4	55.7	600	8908
Lowcarbon steel ^a^	$1.7\times 10^{6}$	0.9	397	1560	7850
FeCo alloy ^b^	$1.8\times 10^{6}$	1.6	100	7000	8120
FeNi alloy ^c^	$1.2\times 10^{6}$	1.1	2.8	190,000	8200

^a^ Properties for C35 standard carbon steel with 0.35% C, 0.15% Si, 0.5% Mn and 0.015% S; ^b^ properties for Vacoflux^®^50, from Vacuumschmelze Gmbh, that are composed of 49% Fe, 49% Co and 2% V; ^c^ properties for Supra50, from Aperam S.A., a Mu-metal that is composed of 51.9% Fe, 47.5% Ni, 0.5% Mn and 0.1% Si.

**Table 2:** Parameters of the robotic arm ^†^: (**a**) the simplified four-bar mechanism as in Figure 12c, and (**b**) the detail of the components of the link $L_{4}$.

(a)				
**Link**	$L_{1}$	$L_{2}$	$L_{3}$	$L_{4}$
$r_{i}$ (mm)	33	444	75	372
$\theta_{i}$	$\theta_{1}$	$\theta_{2}$	$\theta_{3}$	$\theta_{3}$
(**b**)				
**Part**	$L_{4a}$	$L_{4b}$	$L_{4c}$	$L_{4g}$
$r_{i}$ (mm)	100	365	68	60
$\vartheta_{i}$	123°	90°	-	90°

^†^ The robotic arm is made of aluminum material.

**Table 3:** Linear coefficient between magnetic field and input current for each coil.

	Coil1	Coil2	Coil3	Coil4	Coil5	Coil6	Coil7	Coil8
$k_{x}$	0.416	0.4176	−0.2981	−0.3506	−1.3067	0.0102	1.1860	−0.1290
$k_{y}$	−0.5723	0.3588	0.3375	−0.3331	0.0496	1.3262	−0.0875	−1.3181
$k_{z}$	−0.3204	−0.7325	−0.4108	−0.4529	0.1263	0.0760	0.1347	0.1263

**Figure A1:**
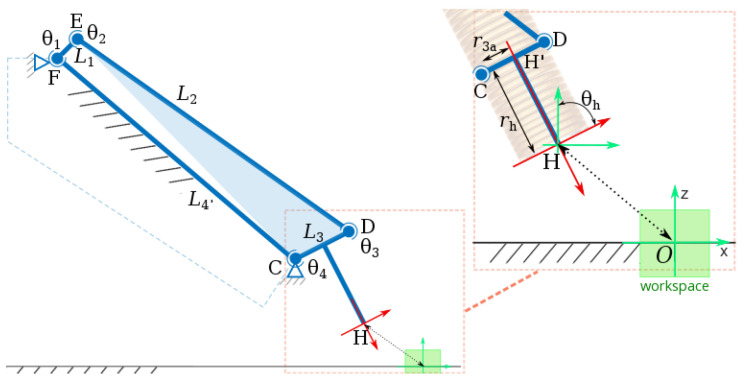
Representation of the robotic arm’s kinematic chain with the four links $L_{1}$–$L_{4}$ and joints $\theta_{1}$–$\theta_{4}$.

**Figure A2:**
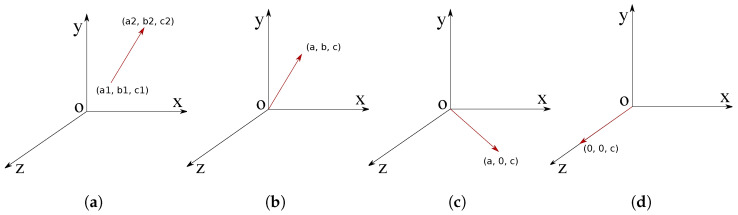
Illustration of rotation step along vectoral axes. A vector refers to a rotational axis in (**a**). (**b**) Translate the rotational axis to the origin coordinate. (**c**) Rotate on the rotational axis to the yoz plane. (**d**) Rotate on the rotational axis to coincide with the *z* axis. Then, the object can be rotated to angle θ on the *z* axis.

**Figure A3:**
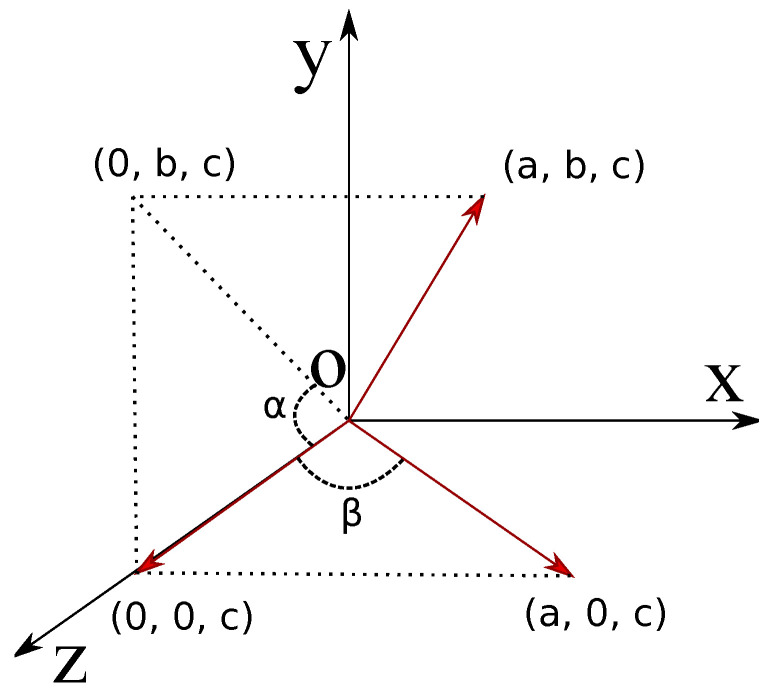
The illustration of the angles between vector axes and coordinate axes.

## Data Availability

The data presented in this study are available from the corresponding authors upon reasonable request.

## Abbreviations

The following abbreviations are used in this manuscript:

DOF	Degree of Freedom
EMA	Electromagnetic Actuation
MIS	Minimally Invasive Surgery
VH	Vitreous Humor
LD	Linear Dichroism
FEM	Finite Element Method
FEA	Finite Element Analysis
PID	Proportional Integral Derivative
DAQ	Data Acquisition
DC	Direct Current

## Appendix A

### Appendix A.1. Coordinate Transformation

In robotics applications, many different coordinate systems can be used to define where robots, sensors and other objects are located [42]. In general, the location of a body in 3D space can be specified by its position and orientation. There are multiple possible representations for these values, some of which are specific to certain applications.

Coordinate transformation is a method of modeling the linkages and joints of a mechanism. Hence, a frame $F_{0}$ is attached to each link of the robotic system. Then, homogeneous transformation is used to describe the spatial relationship between the two adjacent links. Through the sequential transformation, the pose (i.e., the position and orientation) of the end-effector relative to the reference frame $F_{0}$ can be finally derived thereby to establish the kinematic equation of the robotic arm. Commonly, each joint of the robot can only achieve one form of motion, such as rotation or translation. Therefore, the mechanical arm joint either hinged or a sliding joint. For the OctoRob EMA platform, currently, only rotational motion of the robotic arm is defined. (The distance between the electromagnet and the workspace center is also affected by the rotational motion of the motor.) The designed electromagnetic coil is fixed on the end-effector of the robotic arm.

### Appendix A.2. Definition of Transformation Matrices

The poses of the end-effector are obtained by Cartesian space transformation of the joints of the robotic arm. This poses of the end-effector relative to the reference $F_{0}$ can be computed by the given set of joint angles and link lengths. The direct geometric model of the OctoRob robotic arm, recalled in Figure A1, includes here the following steps: First, the pose (*C* and *F*) of the fixed link is characterized in the reference $F_{0}$. Second, the transformation matrix related to the active joint $\theta_{1}$ is established. Thus, the offset angle (namely, the Denavit–Hartenberg parameters) between two adjacent links should be determined. Third, the coordinate transformation can be defined by the product of each transformation matrix, including their rotation and the translation matrices. Last, the all transformation matrices are applied from the fixed pose to obtain the pose of the end-effector *H* in $F_{0}$.

Furthermore, the inverse geometric model means the calculation of each joint angle $\theta_{i}$ of the robotic arm from the given pose of the end-effector described by Cartesian coordinate system. The inverse geometric model is actually multi-solvent—that is, a specific status of the end-effector can be achieved in various combinations of the robotic joint angles, and the solution of inverse geometric is usually difficult to determine.

Finally, the pose accuracy and repeated precision have been considered in the measure. The positioning accuracy is used as an indicator of ability for achieving a Cartesian coordinate system by the provided joint angle of the robotic arm using the direct geometric model. Besides the repeatability, precision presents the capability for manipulation of the end-effector in a given Cartesian coordinate system by using the inverse geometry. Usually, the repeatability precision of the robotic arm is great, and the positioning accuracy is poor. Therefore, such an effect of movement should be considered in the designing process of the robotic arm.

### Appendix A.3. Rotation Matrix

The rotation matrix can be used to rotate the frame linked to an object in Euclidean space. The rotation matrix determines its sign by using a right-hand rule, which is the positive sign produced by counterclockwise rotation along its axis. Let ${\mathrm{Rot}}_{x}\theta$ be a pure rotation about the axis *x* by an angle θ, which is basically defined as:

(A1) $${\mathrm{Rot}}_{x}\theta =\left(\begin{array}{ccc} 1 & 0 & 0\\ 0 & \cos\left(\theta \right) & -\sin\left(\theta \right)\\ 0 & \sin\left(\theta \right) & \cos\left(\theta \right) \end{array}\right)$$

For 3D rotation transformation, it remains to define the pure rotation about the *y* and *z* axes, and we get:

(A2) $$\begin{array}{cc} {\mathrm{Rot}}_{y}\theta & =\left(\begin{array}{ccc} \cos\left(\theta \right) & 0 & \sin\left(\theta \right)\\ 0 & 1 & 0\\ -\sin\left(\theta \right) & 0 & \cos\left(\theta \right) \end{array}\right) \end{array}$$

(A3) $$\begin{array}{cc} {\mathrm{Rot}}_{z}\theta & =\left(\begin{array}{ccc} \cos\left(\theta \right) & -\sin\left(\theta \right) & 0\\ \sin\left(\theta \right) & \cos\left(\theta \right) & 0\\ 0 & 0 & 1 \end{array}\right) \end{array}$$

These basic rotation matrices can apply directly to the general rotations. In the 3D coordinates, the general rotation matrix can be obtained by the following equation:

(A4) $$R\left(\alpha ,\beta ,\gamma \right)={\mathrm{Rot}}_{z}\alpha {\mathrm{Rot}}_{y}\beta {\mathrm{Rot}}_{x}\gamma$$

where α, β and γ represent rotation yaw, pitch and roll angles about axes *z*, *y* and *x*, respectively.

Commonly, it is more convenient to define and utilize global rotation matrix $R\left(\alpha ,\beta ,\gamma \right)$. For instance, when a vector p is rotated with a sole angle θ along a arbitrary vector, the following steps can be used for the computation to obtain the transformation matrix. Let us consider the rotation of a vector given as $p=\left[a_{2}-a_{1},b_{2}-b_{1},c_{2}-c_{1}\right]=\left[a,b,c\right]$, which is illustrated in Figure A2a. In Figure A2b, the rotation axis is translated to the origin coordinate *O* as in step 1. Step 2 is the operation of rotating the rotation axis in the yoz-plane, as shown in Figure A2c. Then, the axis of rotation is changed to coincide with the *z* axis in step 3, as shown in Figure A2d. After finishing the above three steps, the object is rotated θ degrees on the *z* axis. Furthermore, the reverse processes of step 3, step 2 and step 1 should be performed, respectively, to complete this rotation process.

### Appendix A.4. Transformation Matrix of a Pure Translation

These operations can be expressed by the transformation matrix composed of a translation matrix and rotation matrices. The transformation matrix of a pure translation is defined with homogeneous coordinates as:

(A5) $$\mathrm{Trans}\left(a,b,c\right)=\left(\begin{array}{cccc} 1 & 0 & 0 & a\\ 0 & 1 & 0 & b\\ 0 & 0 & 1 & c\\ 0 & 0 & 0 & 1 \end{array}\right)$$

with *a*, *b* and *c* being the translation on the *x*, *y* and *z* axes, respectively. We also use the notation ${\mathrm{Trans}}_{x}\left(a\right)$ to denote the pure translation on the *x* axis by a value *a*.

### Appendix A.5. Homogeneous Transformation Matrices

Let p=[a,b,c] be a vector, as illustrated in Figure A3. The rotation matrix ${\mathrm{Rot}}_{x}\gamma$ is defined for the rotation of the vector’s axis on the *x* axis to the xOz plane; that is:

(A6) $${\mathrm{Rot}}_{x}\theta \left(\gamma \right)=\left(\begin{array}{cccc} 1 & 0 & 0 & 0\\ 0 & \cos\left(\gamma \right) & -\sin\left(\gamma \right) & 0\\ 0 & \sin\left(\gamma \right) & \cos\left(\gamma \right) & 0\\ 0 & 0 & 0 & 1 \end{array}\right)=\left(\begin{array}{cccc} 1 & 0 & 0 & 0\\ 0 & \frac{c}{\sqrt{b^{2}+c^{2}}} & -\frac{b}{\sqrt{b^{2}+c^{2}}} & 0\\ 0 & \frac{b}{\sqrt{b^{2}+c^{2}}} & \frac{c}{\sqrt{b^{2}+c^{2}}} & 0\\ 0 & 0 & 0 & 1 \end{array}\right)$$

The vector axis is rotated on the *y* axis to coincide with the *z* axis on the xOz plane by the following rotation matrix:

(A7) $${\mathrm{Rot}}_{y}-\beta =\left(\begin{array}{cccc} \cos\left(-\beta \right) & 0 & \sin\left(-\beta \right) & 0\\ 0 & 1 & 0 & 0\\ -\sin\left(-\beta \right) & 0 & \cos\left(-\beta \right) & 0\\ 0 & 0 & 0 & 1 \end{array}\right)=\left(\begin{array}{cccc} \frac{\sqrt{b^{2}+c^{2}}}{\sqrt{a^{2}+b^{2}+c^{2}}} & 0 & -\frac{a}{\sqrt{a^{2}+b^{2}+c^{2}}} & 0\\ 0 & 1 & 0 & 0\\ \frac{a}{\sqrt{a^{2}+b^{2}+c^{2}}} & 0 & \frac{\sqrt{b^{2}+c^{2}}}{\sqrt{a^{2}+b^{2}+c^{2}}} & 0\\ 0 & 0 & 0 & 1 \end{array}\right)$$

Let us note that the angle of rotation matrix is here negative due to the vector axis rotating clockwise on the *y* axis. Finally, since the arbitrary rotation axis and *z* axis coincide, the vector can be rotated by angle α on the *z* axis by applying:

(A8) $${\mathrm{Rot}}_{z}\alpha =\left(\begin{array}{cccc} \cos\left(\alpha \right) & -\sin\left(\alpha \right) & 0 & 0\\ \sin\left(\alpha \right) & \cos\left(\alpha \right) & 0 & 0\\ 0 & 0 & 1 & 0\\ 0 & 0 & 0 & 1 \end{array}\right)$$

When the rotation axis is connected to origin *O*, the rotation transformation matrix is computed from step 2, here, without translation. The transformation matrix can be calculated as follows:

(A9) $$T_{\mathrm{rot}}={\mathrm{Rot}}_{x}\left(-\gamma \right){\mathrm{Rot}}_{y}\left(\beta \right){\mathrm{Rot}}_{z}\left(-\alpha \right){\mathrm{Rot}}_{x}\left(\gamma \right)$$

where the matrix T should be used for left multiplication of the vector; that is, $p^{\prime }=T_{\mathrm{rot}}p$. The equations of each step are substituted into the (A9) to obtain the global transformation:

(A10) $$T_{\mathrm{rot}}=\left(\begin{array}{cccc} a^{2}+\left(1-a^{2}\right)\cos\left(\theta \right) & ab\left(1-\cos\left(\theta \right)\right)-c\sin\left(\theta \right) & ac\left(1-\cos\left(\theta \right)\right)+b\sin\left(\theta \right) & 0\\ ab\left(1-\cos\left(\theta \right)\right)+c\sin\left(\theta \right) & b^{2}+\left(1-b^{2}\right)\cos\left(\theta \right) & bc\left(1-\cos\left(\theta \right)\right)-a\sin\left(\theta \right) & 0\\ ac\left(1-\cos\left(\theta \right)\right)-b\sin\left(\theta \right) & bc\left(1-\cos\left(\theta \right)\right)+a\sin\left(\theta \right) & c^{2}+\left(1-c^{2}\right)\cos\left(\theta \right) & 0\\ 0 & 0 & 0 & 1 \end{array}\right)$$

If the vector p is not on the origin *O* of the frame, as in Figure A2a, the first translation and last reverse operation cannot be omitted. The transformation matrix is consequently derived from:

(A11) $$T=\mathrm{Trans}\left(-a_{1},-b_{1},-c_{1}\right)T_{\mathrm{rot}}\mathrm{Trans}\left(a_{1},b_{1},c_{1}\right)$$

where $\left(a_{1},b_{1},c_{1}\right)$ denotes the origin of the vector p. Therefore, the final transformation matrix is expressed as:

(A12) $$T=\left(\begin{array}{cccc} a^{2}+bc\cos\left(\theta \right) & ab\varsigma -c\sin\left(\theta \right) & ac\varsigma +b\sin\left(\theta \right) & \left(a_{1}bc-a\left(b_{1}b+c_{1}c\right)\right)\varsigma +\left(b_{1}c-c_{1}b\right)\sin\left(\theta \right)\\ ab\varsigma +c\sin\left(\theta \right) & b^{2}+ac\cos\left(\theta \right) & bc\varsigma -b\sin\left(\theta \right) & \left(b_{1}ac-b\left(a_{1}a+c_{1}c\right)\right)\varsigma +\left(c_{1}a-a_{1}c\right)\sin\left(\theta \right)\\ ac\varsigma -b\sin\left(\theta \right) & bc\varsigma +a\sin\left(\theta \right) & c^{2}+ab\cos\left(\theta \right) & \left(c_{1}ab-c\left(a_{1}a+b_{1}b\right)\right)\varsigma +\left(a_{1}b-b_{1}a\right)\sin\left(\theta \right)\\ 0 & 0 & 0 & 1 \end{array}\right)$$

with $\varsigma =\left(1-\cos\left(\theta \right)\right)$; $ab=\left(a^{2}+b^{2}\right)$, $ac=\left(a^{2}+c^{2}\right)$ and $bc=\left(b^{2}+c^{2}\right)$.

The resulting homogeneous transformation matrix can be used to obtain the positions and orientations of the electromagnets for the mechanical design of the robotic arms as well.

## References

[1] Chautems C., Zeydan B., Charreyron S., Chatzipirpiridis G., Pané S., Nelson B.J. (2017). Magnetically powered microrobots: A medical revolution underway?. Eur. J. Cardio-Thorac. Surg..

[2] Chen R., Folio D., Ferreira A. (2021). Mathematical approach for the design configuration of magnetic system with multiple electromagnets. Robot. Auton. Syst..

[3] Chen R., Folio D., Ferreira A. (2022). Analysis and Comparison of Electromagnetic Microrobotic Platforms for Biomedical Applications. Appl. Sci..

[4] Kummer M.P., Abbott J.J., Kratochvil B.E., Borer R., Sengul A., Nelson B.J. (2010). OctoMag: An electromagnetic system for 5-DOF wireless micromanipulation. IEEE Trans. Robot..

[5] Gupta P.K., Jensen P.S., de Juan E. (1999). Surgical forces and tactile perception during retinal microsurgery. Proceedings of the International Conference on Medical Image Computing and Computer-Assisted Intervention.

[6] Jagtap A.D., Riviere C.N. Applied force during vitreoretinal microsurgery with handheld instruments. Proceedings of the Annual International Conference of the IEEE Engineering in Medicine and Biology Society.

[7] Singhy S., Riviere C. Physiological tremor amplitude during retinal microsurgery. Proceedings of the IEEE Annual Northeast Bioengineering Conference.

[8] Fine H.F., Wei W., Goldman R.E., Simaan N. (2010). Robot-assisted ophthalmic surgery. Can. J. Ophthalmol..

[9] Pitcher J.D., Wilson J.T., Tsao T.C., Schwartz S.D., Hubschman J.P. (2012). Robotic eye surgery: Past, present, and future. J. Comput. Sci. Syst. Biol..

[10] Spandau U., Scharioth G. (2017). Cutting Edge of Ophthalmic Surgery: From Refractive SMILE to Robotic Vitrectomy.

[11] Channa R., Iordachita I., Handa J.T. (2017). Robotic Eye Surgery. Retina.

[12] Zhang L., Abbott J.J., Dong L., Kratochvil B.E., Bell D., Nelson B.J. (2009). Artificial bacterial flagella: Fabrication and magnetic control. Appl. Phys. Lett..

[13] Zhang L., Abbott J.J., Dong L., Peyer K.E., Kratochvil B.E., Zhang H., Bergeles C., Nelson B.J. (2009). Characterizing the swimming properties of artificial bacterial flagella. Nano Lett..

[14] Zhang L., Peyer K.E., Nelson B.J. (2010). Artificial bacterial flagella for micromanipulation. Lab Chip.

[15] Peyer K.E., Zhang L., Nelson B.J. (2013). Bio-inspired magnetic swimming microrobots for biomedical applications. Nanoscale.

[16] Peyer K.E., Tottori S., Qiu F., Zhang L., Nelson B.J. (2013). Magnetic helical micromachines. Chem. Eur. J..

[17] Nelson B.J., Peyer K.E. (2014). Micro-and nanorobots swimming in heterogeneous liquids. ACS Nano.

[18] Nelson B.J., Kaliakatsos I.K., Abbott J.J. (2010). Microrobots for minimally invasive medicine. Annu. Rev. Biomed. Eng..

[19] Snell R.S., Lemp M.A. (2013). Clinical Anatomy of the Eye.

[20] Tang W.M., Han D.P. (2000). A study of surgical approaches to retinal vascular occlusions. Arch. Ophthalmol..

[21] Shahid H., Hossain P., Amoaku W. (2006). The management of retinal vein occlusion: Is interventional ophthalmology the way forward?. Br. J. Ophthalmol..

[22] Meredith T.A. (2000). Atlas of Retinal and Vitreous Surgery. Retina.

[23] Bonfiglio A., Repetto R., Siggers J.H., Stocchino A. (2013). Investigation of the motion of a viscous fluid in the vitreous cavity induced by eye rotations and implications for drug delivery. Phys. Med. Biol..

[24] Bonfiglio A., Lagazzo A., Repetto R., Stocchino A. (2015). An experimental model of vitreous motion induced by eye rotations. Eye Vis..

[25] Silva A.F., Alves M.A., Oliveira M.S.N. (2017). Rheological Behaviour of Vitreous Humour. Rheol. Acta.

[26] Purcell E.M. (1977). Life at low Reynolds number. Am. J. Phys..

[27] Bekerman I., Gottlieb P., Vaiman M. (2014). Variations in eyeball diameters of the healthy adults. J. Ophthalmol..

[28] Amblard F., Yurke B., Pargellis A., Leibler S. (1996). A magnetic manipulator for studying local rheology and micromechanical properties of biological systems. Rev. Sci. Instrum..

[29] Dogangil G., Ergeneman O., Abbott J.J., Pané S., Hall H., Muntwyler S., Nelson B.J. Toward targeted retinal drug delivery with wireless magnetic microrobots. Proceedings of the IEEE/RSJ International Conference on Intelligent Robots and Systems (IROS).

[30] Abolhassani N., Patel R., Moallem M. (2007). Needle Insertion into Soft Tissue: A Survey. Med. Eng. Phys..

[31] Chen R., Folio D., Ferreira A. Performance metrics for a robotic actuation system using static and mobile electromagnets. Proceedings of the 2019 International Conference on Robotics and Automation (ICRA).

[32] Petruska A.J., Nelson B.J. (2015). Minimum bounds on the number of electromagnets required for remote magnetic manipulation. IEEE Trans. Robot..

[33] Wong D., Steager E.B., Kumar V. (2016). Independent control of identical magnetic robots in a plane. IEEE Robot. Autom. Lett..

[34] Etiévant M., Bolopion A., Régnier S., Andreff N. An Improved Control-Oriented Modeling of the Magnetic Field. Proceedings of the IEEE International Conference on Robotics and Automation (ICRA).

[35] Jing X., Guo W. (2019). Modeling and Configuration Design of Electromagnetic Actuation Coil for a Magnetically Controlled Microrobot. Chin. J. Mech. Eng..

[36] Furlani E.P. (2001). Permanent Magnet and Electromechanical Devices: Materials, Analysis, and Applications.

[37] McCaig M. (1977). Permanent Magnets in Theory and Practice.

[38] Woolman J., Mottram R. (1964). The Mechanical and Physical Properties of the British Standard En Steels.

[39] Kummer M.P. (2010). 5-DOF Wireless Micromanipulation Using Soft-Magnetic Core Electromagnets. Ph.D. Thesis.

[40] Zhang Z., Menq C.H. (2011). Design and Modeling of a 3-D Magnetic Actuator for Magnetic Microbead Manipulation. IEEE/ASME Trans. Mechatron..

[41] Griffiths D.J. (2017). Introduction to Electrodynamics.

[42] Khalil W. (2019). Modeling and Control of Manipulators—Part I: Geometric and Kinematic Models. Ph.D. Thesis.

